# Space- and Time-Resolved Metabolomics of a High-Grade Serous Ovarian Cancer Mouse Model

**DOI:** 10.3390/cancers14092262

**Published:** 2022-04-30

**Authors:** Samyukta Sah, Xin Ma, Andro Botros, David A. Gaul, Sylvia R. Yun, Eun Young Park, Olga Kim, Samuel G. Moore, Jaeyeon Kim, Facundo M. Fernández

**Affiliations:** 1 School of Chemistry and Biochemistry, Georgia Institute of Technology, Atlanta, GA 30332, USA; ssah9@gatech.edu (S.S.); xin.ma@chemistry.gatech.edu (X.M.); david.gaul@chemistry.gatech.edu (D.A.G.); smoore83@gatech.edu (S.G.M.); 2 Departments of Biochemistry and Molecular Biology, Indiana University School of Medicine, Indianapolis, IN 46202, USA; aabotros@iu.edu (A.B.); sylyun@iu.edu (S.R.Y.); epark@truebinding.com (E.Y.P.); olga.kim@nih.gov (O.K.); 3 Indiana University Melvin and Bren Simon Comprehensive Cancer Center, Indianapolis, IN 46202, USA; 4 Institute of Bioengineering and Biosciences, Georgia Institute of Technology, Atlanta, GA 30332, USA

**Keywords:** high-grade serous ovarian cancer, metabolomics, mass spectrometry, imaging, biomarkers

## Abstract

**Simple Summary:**

The underlying mechanisms associated with ovarian cancer progression remain largely unknown, making it one of the most lethal cancers. To understand the disease pathogenesis, our study involved longitudinal serum metabolomics profiling of a triple-mutant mouse model of ovarian cancer that captured the dynamic metabolic response from disease onset until mouse death. These experiments were complemented with spatial lipidomic profiling of the entire reproductive system of the triple-mutant mice, enabling us to visualize the tissue heterogeneity and lipid alterations within tumors. A combined longitudinal and spatial map of metabolomic alterations associated with ovarian cancer progression is presented, serving as a comprehensive guide towards understanding the disease origin and progression.

**Abstract:**

The dismally low survival rate of ovarian cancer patients diagnosed with high-grade serous carcinoma (HGSC) emphasizes the lack of effective screening strategies. One major obstacle is the limited knowledge of the underlying mechanisms of HGSC pathogenesis at very early stages. Here, we present the first 10-month time-resolved serum metabolic profile of a triple mutant (TKO) HGSC mouse model, along with the spatial lipidome profile of its entire reproductive system. A high-coverage liquid chromatography mass spectrometry-based metabolomics approach was applied to longitudinally collected serum samples from both TKO (*n* = 15) and TKO control mice (*n* = 15), tracking metabolome and lipidome changes from premalignant stages to tumor initiation, early stages, and advanced stages until mouse death. Time-resolved analysis showed specific temporal trends for 17 lipid classes, amino acids, and TCA cycle metabolites, associated with HGSC progression. Spatial lipid distributions within the reproductive system were also mapped via ultrahigh-resolution matrix-assisted laser desorption/ionization (MALDI) mass spectrometry and compared with serum lipid profiles for various lipid classes. Altogether, our results show that the remodeling of lipid and fatty acid metabolism, amino acid biosynthesis, TCA cycle and ovarian steroidogenesis are critical components of HGSC onset and development. These metabolic alterations are accompanied by changes in energy metabolism, mitochondrial and peroxisomal function, redox homeostasis, and inflammatory response, collectively supporting tumorigenesis.

## 1. Introduction

Ovarian cancer (OC) patients suffer the highest mortality rate among all gynecological malignancies [1]. For 2020, the Global Cancer Observatory estimated 207,252 ovarian cancer deaths worldwide [2]. In particular, high-grade serous carcinoma (HGSC) is the most common and deadliest OC subtype, accounting for 70–80% of deaths [3]. HGSC is characterized by asymptomatic tumor growth that consequently leads to its diagnosis at advanced stages when the cancer has metastasized. The driving mechanisms for HSGC onset and progression remain largely unknown, and to date there are no effective screening methods available to the general population [4,5]. Symptomatic patients are typically subject to trans-vaginal ultrasound exams, and measurement of serum CA125 levels [5]. However, elevations in CA125 have been reported for many non-ovarian malignancies, and this biomarker exhibits low specificity and sensitivity for early stage OC. The dismally low HGSC survival rate calls for a better understanding of the factors critical to its development, in turn facilitating biomarker discovery for early stages to improve clinical outcomes.

Over the past few years, significant efforts have been made to understand the disease biology of ovarian HGSC. Substantial evidence has emerged indicating the fallopian tube as the site of origin for most ovarian cancers [6], and genomics studies have revealed the critical role of genetic alterations in the development of HGSC, including TP53, PTEN, and BRCA mutations in women with hereditary breast cancer susceptibility [7]. One hallmark characteristic of cancer cells is their ability to rewire metabolism to sustain a favorable environment for tumor growth [8]. As such, metabolomics—the examination of changes in the abundance of metabolites in biological samples—can be leveraged to provide snapshots of the HGSC metabolic phenotype. Examples include metabolomics studies of ovarian cancer in serum [9,10,11,12,13], urine [14] and tissue [15]. Along these lines, our team has characterized the serum metabolome of triple-mutant (TKO) p53 LSL-R172H/+ Dicer1 flox/flox Pten flox/flox Amhr2 cre/+ mice, an animal model that reproduces the clinical metastasis of human HGSC in 100% of cases with close phenotypic, histological, and molecular similarities [16]. This TKO animal is developed by conditionally deleting two critical genes, *Dicer* and *Pten*, and by adding a p53 mutation (R172H), which is equivalent to the p53-R175H mutant found in almost 96% of human HGSC cases, making it a faithful representation of human HGSC. In TKO mice, HGSC originates and develops in the fallopian tube, typically within 10–16 weeks after birth, envelopes the ovaries, and then aggressively metastasizes throughout the abdominal cavity, killing the mice between 24–40 weeks [16]. Serum metabolomics of these animals showed evidence for dysregulated phospholipid, sphingolipid, sterol lipid, 2(3)-hydroxysebacic acids, glycoproteins and glycolipid metabolism [12]. In addition, increasing evidence has shown alterations in fatty acid and lipid [9,10,11,12], nucleotide [12] and amino acid [13] abundances. These metabolomics experiments, however, have been typically performed as a single time-point comparison between disease and control states so the metabolic alterations involved in HGSC progression over time have so far remained unexplored.

Here, we present the first 10-month time-resolved serum metabolomics study of an animal model of ovarian HGSC. Ultrahigh performance liquid chromatography-mass spectrometry (UHPLC-MS) was used for profiling sequentially collected serum samples from TKO mice starting from 8 weeks of age until death and/or ascites. Both the serum lipidome and polar metabolome were profiled using two complementary UHPLC-MS techniques for maximum coverage. Unlike conventional single time-point studies, which do not fully capture the dynamic metabolic response associated with disease development, time-resolved metabolic profiling allowed mapping metabolite alterations as the HGSC progressed. To complement UHPLC-MS analysis, and to understand the origin of the alterations observed in serum, we also performed tissue spatial lipidomics using matrix-assisted laser desorption/ionization (MALDI) ultrahigh-resolution Fourier Transform Ion Cyclotron Resonance (FTICR) mass spectrometry imaging (MSI) [17]. MSI experiments allowed us to connect lipid spatial distributions in reproductive system tissues with changes in serum. To our knowledge, this study represents the most in-depth analysis of the mouse ovarian cancer metabolome achieved to date.

## 2. Materials and Methods

### 2.1. Triple Mutant (TKO) Mice

p53LSL R172H/+ Dicer1flox/flox Ptenflox/flox Amhr2cre/+ mice were generated by mating p53LSL-R172H/+Dicer1flox/floxPtenflox/flox female mice with Dicer1flox/floxPtenflox/floxAmhr2cre/+ male mice. p53LSL-R172H/+Dicer1flox/floxPtenflox/flox mice were used as TKO controls (ctrl). TKO ctrl mice carry the same genetic background as TKO mice but do not develop HGSC. p53LSL R172H/+ Dicer1flox/flox Ptenflox/flox Amhr2cre/+ mice were sacrificed in accordance with the animal protocol (21124) approved by the IACUC at Indiana University School of Medicine (Indianapolis, IN, USA).

### 2.2. Sequential Blood Sampling

Blood samples were collected from 17 TKO mice and 16 TKO ctrl mice starting at 8 weeks of age. A sequential blood sampling procedure was conducted and samples from each mouse were collected every two weeks until humane end point for sacrifice or development of ascites. Two TKO mice died after 14 and 16 weeks of age and were not included in the UHPLC-MS analysis. One TKO ctrl mouse died after 10 weeks of age and was also excluded.

### 2.3. Chemicals

For metabolomics experiments, LC-MS grade methanol, LC-MS grade water, LC-MS grade acetonitrile, LC-MS grade 2-propanol, formic acid (99.5+%), ammonium acetate, ammonium formate and ammonium hydroxide were purchased from Fisher Chemical (Fisher Scientific International, Inc., Pittsburgh, PA, USA) and used to prepare chromatographic mobile phases. Isotopically labeled lipid standards (Table S1a) were purchased from Avanti Polar Lipids (Alabaster, AL, USA) and used to prepare the lipid internal standard mixture. Isotopically labeled polar metabolite standards (Table S1b) were purchased from Cambridge Isotope Laboratories (Tewksbury, MA, USA) and were used to prepare the internal standard mixture for HILIC experiments.

For spatial lipidomics experiments, carboxymethyl cellulose (CMC), gelatin from bovine skin (Type B), 1,5-naphthalenediamine (1,5-DAN, ≥97%) and isopentane (≥95%) were purchased from Sigma Aldrich (St. Louis, MO, USA) and used as received. Acetonitrile (HPLC grade), methanol (HPLC grade) and chloroform (HPLC grade) were purchased from VWR and also used as received.

### 2.4. UHPLC-MS Serum Metabolomics

Reverse phase (RP) ultra-high-performance liquid chromatography, mass spectrometry (UHPLC-MS) and hydrophilic interaction liquid chromatography (HILIC) UHPLC-MS analysis were performed to obtain a deeper coverage of the metabolome. Serum samples were thawed on ice, followed by metabolite extraction of both non-polar (lipid) and polar metabolites using two different sample preparation protocols, detailed in the supplementary information. Reverse phase chromatography was performed with a Thermo Accucore C30, 150 × 2.1 mm, 2.6 µm particle size column, and hydrophilic interaction liquid chromatography (HILIC) chromatography was performed with a Waters ACQUITY UHPLC BEH Amide 150 × 2.1 mm, 1.7 µm particle size column. A Q Exactive HF Orbitrap mass spectrometer (ThermoFisher Scientific, Waltham, MA, USA) was used for RP UHPLC-MS analysis, and an Orbitrap ID-X Tribrid mass spectrometer (ThermoFisher Scientific) was used for HILIC UHPLC-MS analysis. Samples were kept at 4 °C in the autosampler during runs, and injection volumes of 2 µL and 1 µL were used for RP and HILIC methods, respectively. Chromatographic gradients, relevant MS parameters and details on MS/MS experiments for metabolite annotation are described in the Supplementary Information (Tables S2 and S3).

### 2.5. Data Processing and Statistical Analysis of LC-MS Datasets

Spectral features (retention time, *m*/*z*) pairs were extracted using Compound Discoverer v3.1. The procedure involved retention time alignment of chromatographic peaks, peak picking, peak area integration, and compound area correction using a quality control (QC)-based regression curve. Chromatographic peaks that were present with less than five times the baseline abundance (peak area of the sample blank) were marked as background signals and removed from the dataset. Furthermore, features that were not present in at least 50% of the QC sample injections or had a relative standard deviation greater than 30% in the QC injections were also removed. All annotated features from the RP dataset, and all features from the negative ion mode HILIC dataset were kept for further analysis. Prior to multivariate data analysis, the quality of pooled QC runs was evaluated (detailed in Supplementary Information). Features with a Benjamini Hochberg adjusted *p*-value lower than 0.05 (*q* < 0.05) were selected as statistically significant based on a two-tailed Welch’s *t*-test and used to map time-resolved metabolic changes associated with ovarian HGSC.

### 2.6. Genetic Algorithms Variable Selection

A panel of metabolites was selected using the genetic algorithm [18] (GA) feature selection method (GA, MATLAB R2020b, The Mathworks, Natick, MA with PLS_Toolbox v.8.1.1, Eigenvector Research, Wenatchee, WA, USA). GA-selected features with the lowest root-mean-square error of cross validation (RMSECV) were used to build orthogonal Partial Least Square Discriminant Analysis (oPLS-DA) models for sample class discrimination. Data were autoscaled prior to oPLS-DA analysis and were cross validated using a 10-fold Venetian blind method.

### 2.7. Lipid Ratio Biomarkers

The diagnostic potential of ratios between two lipid species was also explored. Data for all statistically significant features (*p* < 0.05) between TKO and TKO ctrl samples were uploaded to the MetaboAnalyst [19] web portal, and the ratios between all possible metabolite pairs calculated. The top 20 metabolite ratios were generated based on *p*-values. The selected ratios were evaluated with a 10-fold cross validated logistic regression model. The best lipid ratio biomarkers were selected based on their *p*-values, area under curve value (AUC), sensitivity, and specificity values obtained from their respective logistic regression models. Data were G-log transformed and autoscaled in the Metaboanalyst portal prior to analysis.

### 2.8. Tissue Spatial Lipidomics

For MS imaging experiments, TKO mice were sacrificed at advanced cancer stage and their reproductive systems were collected and stored at −80 °C. Following examination of a variety of tissue samples, we focused on a TKO mouse reproductive system that showed a HGSC on one of the fallopian tubes, with the healthy ovary engulfed in the tumor and adjacent cysts. Tissue preparation for imaging experiments is described in the Supplementary Information.

A Bruker SolariX 12-Tesla Fourier-transform ion cyclotron resonance (FTICR) mass spectrometer equipped with a MALDI ion source was used for all spatial lipidomics imaging experiments. The mass spectrometer was operated in the negative ion mode for fatty acid and lipid feature discovery in the 150–1200 *m*/*z* range. Detailed FTICR conditions are listed in the Supplementary Information. Observed ions in the average mass spectrum were subject to Lipid Maps and HMDB database searches using METASPACE [20]. Features with a false discovery rate of 10% or less were chosen and compared to features annotated in UHPLC-MS serum studies.

### 2.9. Time Course Data Alignment

Variability in the time-course of HGSC, arising from individual differences between animals, was observed in our time-series metabolomics dataset (Figure S1). To better align the data, we created a “percent lifetime” variable, which is the normalized time until death for each mouse. Percent lifetime for each animal was calculated according to the following equation:

$$\%Lifetime=\frac{Age\;of\;mouse}{Total\;lifespan\;or\;the\;age\;at\;the\;last\;time\;point\;of\;blood\;collection\;}\;\times \;100\%$$

The age of all mice in weeks and their corresponding %lifetime values are given in Supplementary Tables S4 and S5. Based on these values, and to simplify time-course data analysis, the dataset was divided into four subgroups: the 20–36 %lifetime subgroup was assigned to a premalignant HGSC stage, the 37–60 %lifetime group was considered early stage I (ET-I, most likely corresponding to tumor onset), 61–80 %lifetime was named early stage II (ET-II, corresponding with early stage tumors with no evident signs of metastasis), and 81–100 %lifetime was assigned to advanced stage (AT) HGSC, where the tumor had metastasized (Figure S2). These stage estimates were made based on our initial study of HGSC in the same TKO mouse model [12].

## 3. Results

### 3.1. HGSC Lipidome Alterations

A total of 11,384 and 3475 de-isotoped and de-adducted “compounds” were extracted from positive and negative ion mode reverse phase (RP) UHPLC-MS data, respectively. Compounds detected in both ion modes were combined, and a total of 1002 species that included fatty acids, glycerophospholipids, lysoglycerophospholipids, carnitines, glycerolipids, sphingolipids, sterol lipids, and some retained polar metabolites such as arginine, citrulline, and ribose-5-phosphate were successfully annotated using MS/MS in-house databases. Typical raw chromatographic data showing separation of various lipid classes is depicted in Figure S3, showcasing the excellent resolution obtained. From the 1002 annotated features, 420 had false-discovery rate (FDR) corrected *p*-values lower than 0.05 (*q* < 0.05) when comparing TKO and TKO ctrl groups (Table S6). To further investigate differences between TKO and TKO ctrl at the lipidome level, the dataset was subject to unsupervised principal component analysis (PCA) using the 420 statistically significant features. PCA showed minimal separation between TKO and controls for serum samples collected in 20–80 %lifetime, whereas TKO samples at 81–100 %lifetime were better separated from the controls (Figure S4a). This was expected due to the complexity of time-series data, indicating that metabolome differences become more pronounced with HGSC progression, as expected. This dataset was further explored using supervised orthogonal partial least squares-discriminant analysis (oPLS-DA). In this case, good clustering between TKO and TKO ctrl was observed on the first two latent variables (Figure S4b), with the first latent variable capturing most of the inter-class variance. Performance characteristics of this oPLS-DA model were 82.5%, 89.0%, and 85.7% cross-validated sensitivity, specificity, and accuracy, respectively.

**Figure 1:**
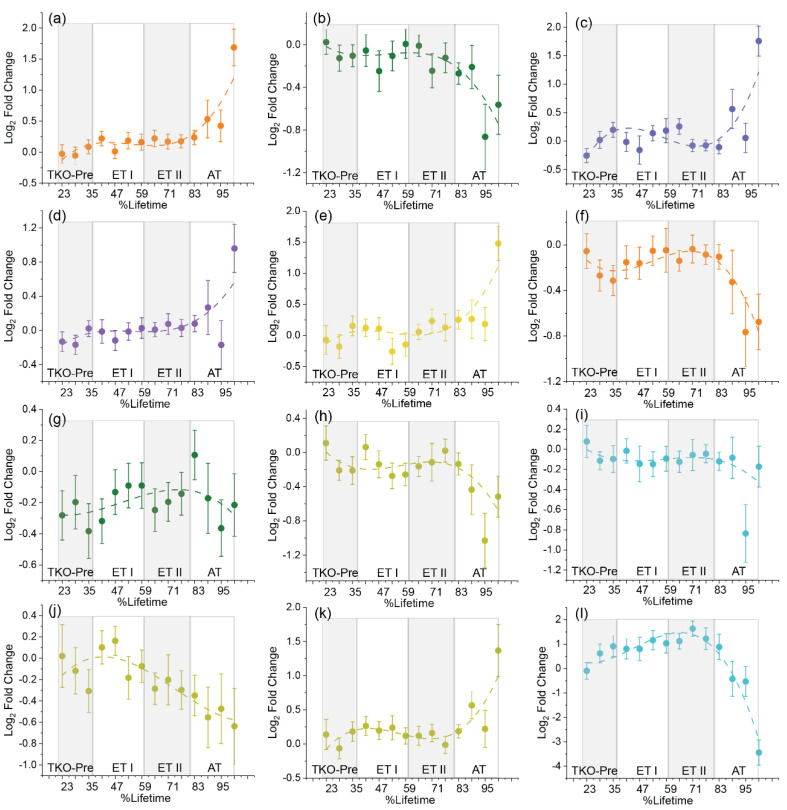
Time−resolved serum lipidomics. Serum abundance fold-changes as a function of %lifetime for (**a**) Ceramides (Cer) (**b**) Sphingomyelins (SM) (**c**) Hexosylceramides (HexCer) (**d**) Phosphatidylcholines (PC) (**e**) Phosphatidylethanolamines (**f**) Lysophosphatidylcholines (LPC) (**g**) Lysophosphatidylethanolamines (LPE) (**h**) Ether PE (**i**) Ether PC (**j**) Short-chain fatty acids (SCFA, carbon chain shorter than 20C) (**k**) very long-chain fatty acids (carbon chain longer than 22C) (**l**) 20 alpha-hydroxyprogesterone. The total abundance for each lipid class was calculated by averaging the relative abundances of all statistically significant lipid species in that class. The *x*-axis shows the %lifetime, calculated as the ratio of the age of mouse at a given serum sampling time point compared to the total lifespan of that specific mouse. The *y*-axis shows the fold change calculated as the base 2 logarithm of the lipid content ratios of TKO/TKO ctrl samples. Positive values indicate higher serum levels in TKO animals, and negative values indicate lower serum levels in TKO animals compared to TKO ctrl. Error bars represent the standard error of the log_2_ fold change between TKO and control samples. HGSC stage estimate at different %lifetimes are shown. TKO-pre corresponds to premalignant stages, ET-I is early stage 1, ET-II is early stage II and AT is advanced stage.

To evaluate the lipidomic profile associated with HGSC development and progression over time, the RP UHPLC-MS dataset was analyzed on a lipid class basis (Figures S5 and S6). Overall, results showed a clearly dysregulated lipidome profile. In early disease, ceramides (Cer) and hexosylceramides (HexCer) showed subtle increases in abundance, while sphingomyelins (SM), lysophosphatidylcholines (LPC), lysophosphatidylethanolamines (LPE), and most ether phosphatidylethanolamines, (PE O-), ether phosphatidylcholines (PC O-) and ether phosphatidylserines (PS O-) decreased (Figures S5 and S6). To characterize the temporal trajectories of these lipid changes, we examined the time-resolved average lipid abundances in TKO mice across the 20–100%lifetime range (8–40 weeks, including premalignant stages). Temporal trends for 17 lipid classes that correlated with disease progression were clearly observed (Figure 1 and Figure S7). SM showed an overall decrease in abundance in TKO mice, whereas Cer, the hydrolysis product of SM, showed an increasing trend (Figure 1a,b, Figures S5 and S6). Phosphatidylcholines (PC), phosphatidylethanolamines (PE), phosphatidylserines (PS) and phosphatidylinositols (PI) showed an overall increasing temporal trend, although for some PC, PE, and PI species the trend was less clear (Figure 1d,e and Figure S7a,b). In contrast, LPC and LPE—the degradation products of PC and PE—decreased in TKO mice, following a characteristic inverted U-shaped time-course trajectory (Figure 1f,g). The ether phospholipids, including all PE O-, most PC O- species, and PS O-species, also decreased in TKO mice over time (Figure 1h,i and Figure S7c). Most triglycerols (TG) and all diglycerols (DG) species showed an increasing temporal trend (Figure S7d,e). Temporal trends for fatty acids (FA) varied based on the carbon chain length. FA with 22 or more carbon atoms in their chains, except FA (28:0), increased in TKO mice over time (Figure 1j,k and Figure S6). FA with carbon chains shorter than 20 carbons were lower in TKO mice and decreased in abundance over time (Figure 1h). Interestingly, the 20-carbon chain FA did not have a specific trend. Similarly, temporal trends for carnitines (Car) were less clear (Figure S7f,g). Two stigmasterol esters, SiE(20:4) and SiE(22:6), were also identified in the dataset and showed an overall decreasing abundance with time in TKO mice (Figure S7h). Sterol lipids, including cholesterol, cholesterol esters, and desmosterol, showed an increased abundance in TKO mice. (Figure S7i). Among all annotated lipid species in the RP datasets, 20 alpha-hydroxyprogesterone (20α-OHP), the reduced form of progesterone, showed the largest increase (log2 fold change = 0.99) at 37–80 %lifetime. In the advanced stages, however, 20 OHP levels decreased in TKO mice (85–100 %lifetime log2 fold change −0.43) (Figure 1l and Figure S8). Overall, significant remodeling of the serum lipidome was observed with disease progression, with more subtle changes seen in the earlier disease stages (20–80 %lifetime), which were amplified in the late disease stage (81–100 %lifetime). These results parallel what is clinically observed for human ovarian cancer patients: that is, asymptomatic disease in early stages that is more easily detectable at late stages.

### 3.2. Alterations in the Abundances of Polar Metabolites

From the negative ion mode HILIC data, a total of 1988 compounds were extracted. These included amino acids and their derivatives, nucleic acids, hydroxy fatty acids, some lysoglycerophospholipids, phytochemicals, and other small organic molecules. From these, 221 compounds had a *q*-value < 0.05. To investigate the metabolomic differences between TKO and TKO ctrl, an exploratory PCA model was built using those 221 features (Figure S9a). Similar to the RP dataset, some overlap between early disease stages (20–80 %lifetime) and TKO controls was observed. Samples from advanced disease stages (81–100 %lifetime), as expected, were better separated from controls. A supervised oPLS-DA model using the 221 HILIC features showed better class clustering (Figure S9b), which could likely be improved by feature selection approaches. Performance characteristics for this oPLS-DA model were 77.5%, 83.4%, and 80.45% cross-validated sensitivity, specificity and accuracy, respectively. To look at the earliest stages of the disease, we focused on features with *q* < 0.05 between TKO and TKO ctrl in the 37–60% lifetime group. Forty-six significant features were used to build an oPLS-DA model (Figure S10). Performance characteristics of this oPLS-DA model were 84.7%, 69.0% and 76.8% sensitivity, specificity, and accuracy, respectively. These features showed acceptable classification power and were chosen for further analysis.

**Figure 2:**
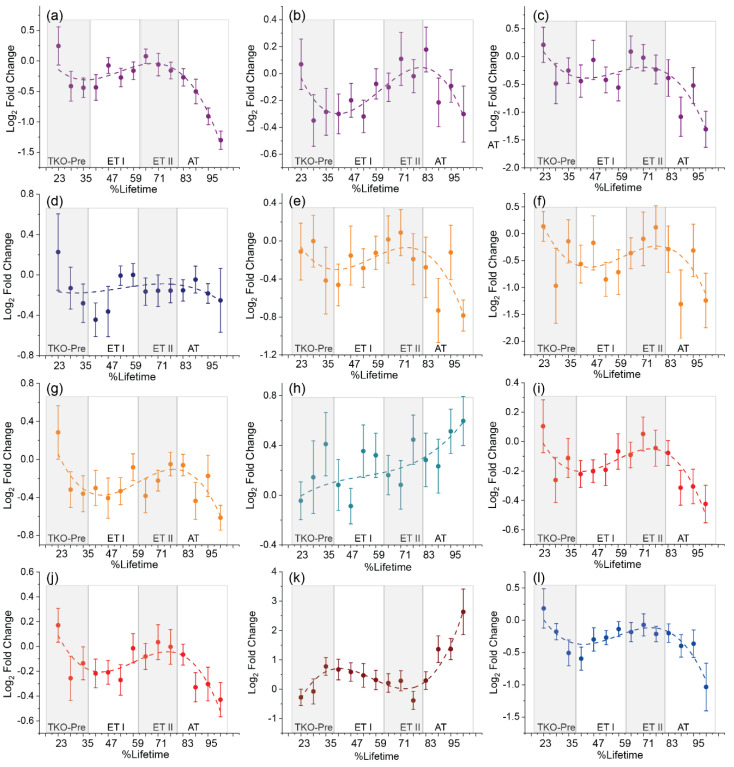
Time−resolved serum metabolomics results for select polar metabolites. Serum abundance fold-changes as a function of %lifetime for (**a**) serine (**b**) threonine (**c**) oxobutanoate (**d**) tryptophan (**e**) oxoglutaric acid (**f**) malic acid (**g**) citric/isocitric acid (**h**) N-acetylglutamine (**i**) citrulline (**j**) ornithine (**k**) bile acids and derivatives and (**l**) arachidonic acid metabolites. The *x*-axis shows the %lifetime, calculated as the ratio of the age of mouse at a given serum sampling time point compared to the total lifespan of that specific mouse. The *y*-axis shows the fold change calculated as the base 2 logarithm of the average abundance ratios between TKO and TKO control samples. Positive values indicate higher serum levels in TKO animals, and negative values indicate lower serum levels in TKO animals compared to TKO control. Error bars represent the standard error of the log_2_ fold change between TKO and ctrl samples. HGSC stage estimate at different %lifetimes are shown. TKO-pre corresponds to premalignant stages, ET-I is early stage 1, ET-II is early stage II and AT is advanced stage.

Metabolite annotation was attempted for these 46 compounds (Table S7), with a total of 35 being annotated with confidence levels ranging from 2 (exact mass and MS/MS spectra match) to 3 (exact mass match). Ten out of these 35 annotated compounds were amino acids, amino acid derivatives, and TCA cycle metabolites (serine, threonine, citrulline, ornithine, tryptophan, N-acetylglutamine, oxobutanoate, citric/isocitric acid, malic acid, and 2-ketoglutaric acid). The remaining 25 compounds included fatty acids, hydroxy fatty acids, a lysoglycerophospholipid LPC(18:2), bile acids and derivatives, terpenes and other phytochemicals. Of these 46 compounds, 42 were decreased in TKO animals, while only 4, the two bile acids, N-acetylglutamine, and an unidentified compound at *m*/*z* 129.9365, were increased in TKO samples (Table S7). The exact origin of the identified phytochemicals is uncertain. However, altered phytochemical metabolism was also found in our previous metabolomics study of a double knockout Dicer1-Pten mouse model of HGSC [11], indicating that the observed alterations in terpenes and isoprenoids could in fact be a useful signature of ovarian HGSC. Nevertheless, sample class discrimination using only the identified amino acids and derivatives, TCA cycle metabolites, bile acids and arachidonic acid metabolites provided 83.1% specificity, 69.0% sensitivity, and 76.05% accuracy in a 2-latent variable oPLS-DA model (Figure S11a). Annotations for these metabolites are given in Table 1. Time-course abundances of amino acids, their derivatives and TCA cycles metabolites showed a characteristic pattern (Figure 2) with decreasing abundance in TKO mice between 30–60 %lifetime, followed by increasing abundance between 61–80 %lifetime, and decreasing abundance between 81–100 %lifetime. This characteristic pattern suggested an increased reliance on these metabolites during the tumor onset and advanced stages of HGSC. Only N-acetylglutamine showed an overall increasing abundance in TKO mice (Figure 2h, Table 1). Bile acids (Figure 2k) showed an interesting increase at the TKO premalignant stages (20–36 %lifetime), a decreasing trend in the early disease stages (36–80 %lifetime), followed by an increase in the advanced stages.

### 3.3. HGSC Discriminant Metabolite Panels

As accurate early detection of HGSC is crucial in improving the clinical outcome, we focused on selecting a smaller discriminant metabolite panel for TKO and TKO ctrl samples in the 37–60 %lifetime, corresponding with HGSC onset stages. Data from both RP and HILIC were combined and subject to genetic algorithm [18] (GA) variable selection (PLS_Toolbox v.8.1.1, Eigenvector Research). However, GA-selected features from the HILIC dataset included some likely exogenous metabolites such as dihydroxybenzene, hydroxylated FA(16:3), and some unknowns, which lowered the biological relevance of such panel. Therefore, we performed GA feature selection only on the statistically significant compounds from the RP dataset. A panel of 22 lipids was selected (Table 2) and used to build a 3-latent variable oPLS-DA model. This model discriminated TKO and TKO ctrl samples with 96.3%, 93.6%, and 95.0% cross-validated specificity, sensitivity, and accuracy, respectively (Figure S11b). To test for any overfitting, permutation testing with 1000 iterations was conducted, and a *p*-value < 0.001 was obtained using group separation distance [21]. These results suggested the oPLS-DA model with 22 lipids was not significantly overfit. Lipids in the panel included several phospholipids with C18:2, C18:0, and C20:4 fatty acid alkyl chain compositions, sphingolipids, glycerolipids, fatty acids including a very long chain fatty acid FA(28:0) and a long chain lysophosphatidylcholine LPC(24:2), citrulline, and (20α-OHP). Furthermore, we evaluated the performance of this 22-feature panel in discriminating TKO and TKO ctrl samples in the 61–100 %lifetime group (ET-II and AT advanced disease stages). The oPLS-DA model discriminated TKO and TKO ctrl in 61–100 %lifetime with 89.7%, 92.2%, and 90.9% cross-validated sensitivity, specificity, and accuracy, respectively, indicating very good performance for this biomarker panel.

### 3.4. Discriminant Lipid Ratios

Often ratios between two or more metabolites carry more information compared to the individual metabolites alone [22]. Ratios, especially between a lipid species and its product, can not only provide high classification accuracy, but can also reveal specific pathway alterations [23]. Here, too, we evaluated the potential of lipid ratios as biomarkers of HSGC. Interestingly, the best performing ratios were seen for the ratios of ceramide(d34:1) and various PE O- (Table 3(a), Figure S12). The top five ratio pairs had AUC values > 0.80. Specificity and sensitivity values were >0.72. Performance of these lipid ratios was further evaluated for samples at the tumor onset stages (ET-I, 37–60 %lifetime) (Table 3(b)). All five lipid ratios were statistically significant and had AUC values greater than 0.70, suggesting that lipid ratio biomarkers could serve as simple indicators for potential tumor onset. The selected lipid ratios also indicated a potentially important pathway association between sphingolipid and ether phospholipid metabolism in HGSC. Although, ratios between ceramides and ether phospholipids abundances have been previously reported as potential biomarkers of ovarian cancer [24], the biological rationale linking sphingolipid and ether phospholipid metabolism is not well developed. A recent study revealed metabolic co-regulation of ether lipids and sphingolipids, suggesting the two lipid classes share biochemical aspects and regulate intracellular transport of glycosylphosphatidylinositol-anchored proteins (GPI-AP) [25], a specialized protein post-translation modification that has been associated with many cancers [26]. Additionally, the repeated selection of Cer(d34:1) as the numerator of the chosen ratios suggests that this ceramide may be an important HGSC biomarker. Intriguingly, increased abundance of Cer(d34:1) has been repeatedly reported in several ovarian cancer studies, including human patients [9,27].

### 3.5. MALDI-MSI Spatial Lipidomics

To determine spatial distributions of lipid metabolites, two TKO and one TKO control reproductive system tissues were cryosectioned and imaged with MALDI-FTICR MSI in negative ion mode. The TKO tissue samples were obtained from TKO mice with advanced stage disease, including primary fallopian tumors and peritoneal metastases, while control reproductive tissues from a TKO control mouse (p53 LSL-R172H/+ Dicer1 flox/flox Pten flox/flox Amhr2 +/+) with no sign of abnormalities. We performed a minimum of three technical replicates from adjacent tissue sections to ensure the repeatability of our methods. Among all replicate measurements, ~140 lipid features were putatively annotated for TKO tissues, whereas ~160 features were putatively annotated in controls (≤10% FDR). The annotated features belonged to 13 lipid classes. Some lipids from these classes, such as cyclopropane fatty acids (CPA), phosphatidic acids (PA), ceramide phosphates (CerP), and phosphatidylglycerols (PG), were not detected in the serum (Figure S5). In order to unambiguously assign the lipid classes so that their alterations can be directly compared to the serum results, we conducted MS/MS experiments and matched fragment ions to the ALEX123 lipid calculator [28]. Assignment of carbon chain length and double bond positions was not possible based on this type of collision-induced dissociation (CID) MS/MS results.

**Figure 3:**
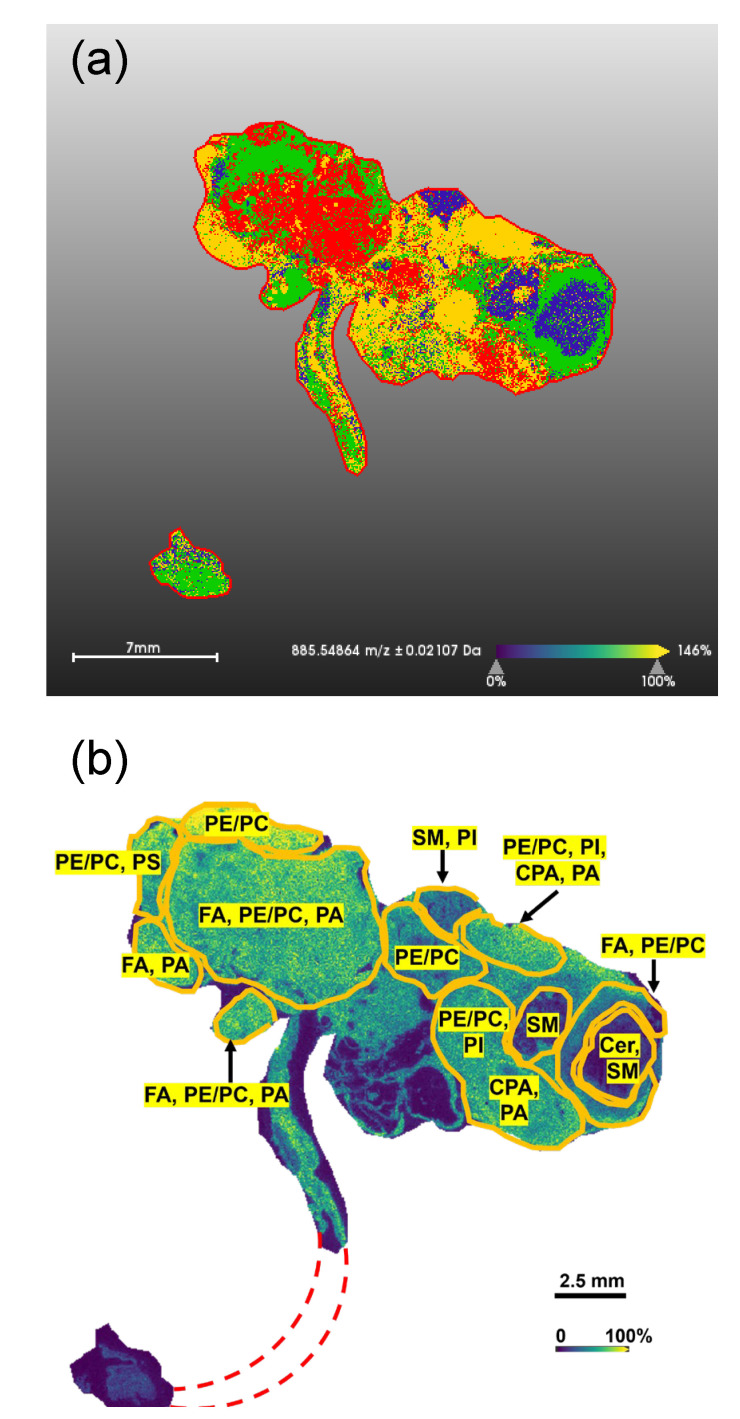
Spatial lipidomics results for the reproductive system of a TKO animal. (**a**) Spatial segmentation of the TKO tissue using a bisecting k-means algorithm, where major sub-regions of the tumor can be identified (labelled in Figure 4). (**b**) Overall spatial distribution of putatively annotated lipid features in the tumor region of the TKO tissue. Circled sub-regions are labeled with certain lipid species that have significant alterations (increased or decreased) compared to control tissues. The red dashed lines indicate the missing uterus due to imperfect tissue sectioning. FA: fatty acids, PE: phosphatidylethanolamines, PC: phosphatidylcholine, PE O-: ether phosphatidylethanolamines, PI: phosphatidylinositols, PS: phosphatidylserines, Cer: ceramides, SM: sphingomyelins, CPA: cyclopropane fatty acids, PA: phosphatidic acids.

**Figure 4:**
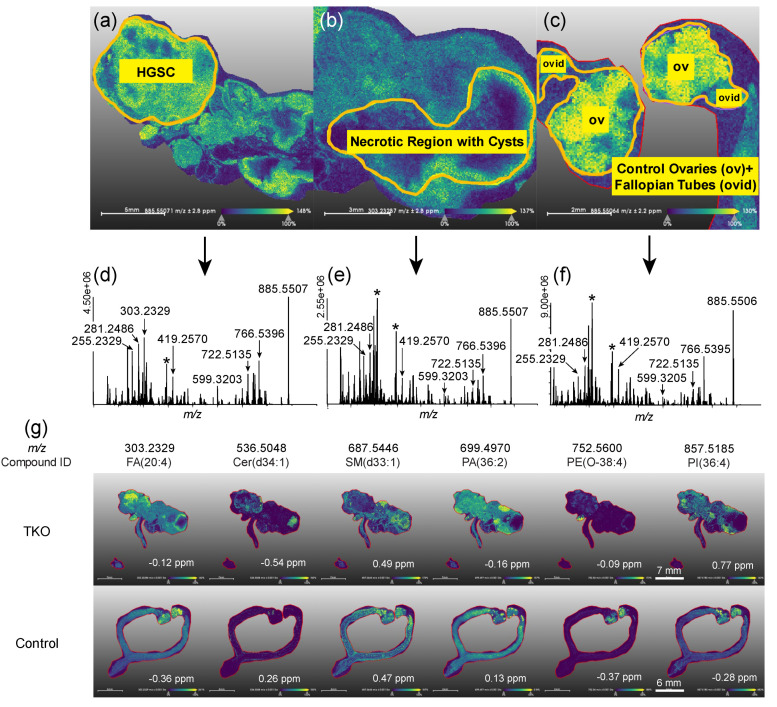
Spatial lipidomics results for specific tissue regions and lipids in TKO and TKO control animals. Average MALDI images of (**a**) HGSC region in TKO animal, (**b**) necrotic TKO tissue region in TKO animal, (**c**) healthy ovaries and fallopian tubes in a TKO control animal. Panels labelled (**d**–**f**) show the corresponding average negative ion mode MALDI mass spectra. Peaks labelled with asterisks (*) are matrix ions in the background mass spectrum. Panel (**g**) shows MALDI images for selected lipid features in TKO and TKO ctrl tissues. The mass errors for the monoisotopic [M−H]^−^ ions are listed at the bottom right of each image. All the ions’ signals were normalized to the total ion current in each image. The mass window used for creating selected ion images was ±0.001 Da.

The MSI dataset was analyzed by spatial segmentation, in which MALDI mass spectra collected for each pixel were clustered based on their spectral similarity using a bisecting k-means algorithm [29]. The segmentation image was color-coded based on the distinct sub-regions identified by the algorithm (Figure 3a), which mapped onto the regions of different lipid abundances shown in Figure 3b. Based on Figure 3b, most annotated lipids were unevenly distributed throughout the entire tumor region. Figure 4a,b show the sub-regions where specific lipid classes were significantly altered. The tumor mainly consisted of three sub-regions: a relatively homogeneous HGSC region, a heterogeneous necrotic region with cysts, and a healthy ovary wrapped by the HGSC. For each of these sub-regions, the average MALDI images and spectra are shown in Figure 4d–f. MALDI images of specific lipid ions were created (see examples in Figure 4g) to study lipid distributions and alterations in the HGSC region relative to the ovaries and fallopian tubes in TKO ctrl tissue, and to compare the results to serum lipid abundances.

Overall, lipid alterations in tissues were far more drastic than those measured in serum (Figure S5), with log2 fold changes that were two to seven-times greater on average. This finding may be explained by the immediate, direct impact of tumor development on the tissue, which leads to more pronounced alterations of the lipids in the specific sub-region (ovaries or fallopian tubes) as a response to tumor progression. For sphingolipids, the same trend as in serum was observed when the HGSC region was compared to the ovaries of the control tissue, i.e., decreased SM and increased Cer levels. However, when compared to the healthy fallopian tubes in the control tissue, both Cer and SM levels were lower in the HGSC region. The decrease in Cer may be due to the pro-mitogenic nature of cancer that induces the cell division (mitosis) or increase the cell division rate [30]. Next, in agreement with the serum lipidome profile, we observed reduced levels of all LPE species and most PE O- species in the HGSC region (Figure S5). PC and PE did not show any clear trends. Likewise, no clear trends for FA and for PA were observed. PS and Phosphatidylinositol (PI) were lower in the HGSC region compared to the healthy fallopian tube. Although we observed an overall increase in PS and PI lipids in serum (Figure S5), time-resolved analysis indicates that levels of both PS and PI fluctuate over time (Figure 1g) with a negative log2 fold change in at 85–95 %lifetime and a positive log2 fold change at time of death (100 %lifetime). Nevertheless, alterations in PI levels, indicative of a dysregulated PI3K/AKT pathway, have been frequently observed in ovarian cancer patients [31].

## 4. Discussion

**Figure 5:**
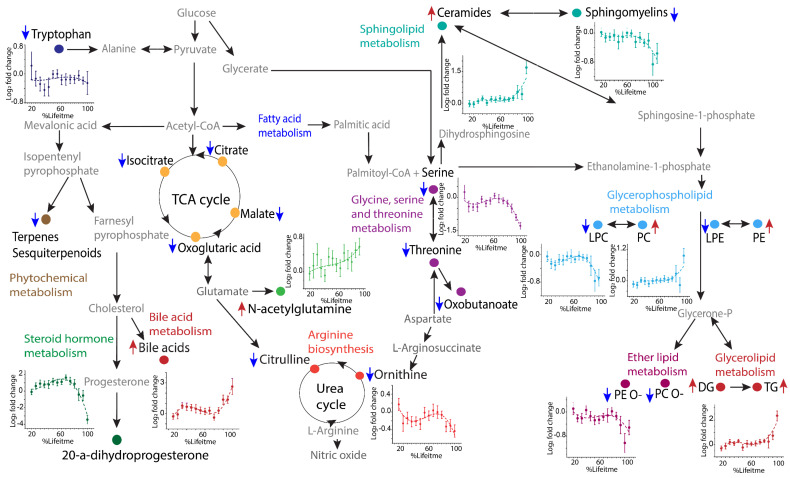
Pathway analysis showing key metabolic alterations observed in TKO mice. Metabolites and lipid classes associated with HGSC development are represented as solid symbols and colored based on their corresponding pathway. Intermediates connecting the pathways are shown in grey text. Blue arrows pointing downwards indicate the metabolite showed an overall decrease in TKO animals compared to TKO controls while the red arrows pointing upwards indicate the metabolite increased in TKO animals. Pathway information was derived from the Kyoto Encyclopedia of Genes and Genomes, MetaboAnalyst, Lipid Pathway Enrichment Analysis, and existing scientific literature. Abbreviations: DG: Diacylglycerols, TG: Triacylglycerols, PC: Phosphatidylcholines, PC O-: Ether phosphatidylcholines, PE: Phosphatidylethanolamines, PE O-: Ether phosphatidylethanolamines, LPE: Lysophosphatidylethanolamines and LPC: Lysophosphatidylcholines.

Although past metabolomics studies have successfully provided evidence of metabolism dysregulation associated with ovarian HGSC, the disease biology and the underlying mechanisms associated with disease progression still remain largely unknown from a metabolomics perspective. Our present study provides a unique opportunity to track the disease metabolic trajectory in animals, as well as visualize the spatial lipidome distributions in HGSC tumors, thereby providing a more in-depth understanding of metabolic remodeling associated with ovarian cancer development and progression. An HGSC metabolism map was constructed by compiling results from analyses of the Kyoto Encyclopedia of Genes [32], Metabolomics Pathway Analysis (MetPA, MetaboAnalyst portal) [33], Lipid Pathway Enrichment Analysis (LIPEA) [34] and various literature references (Figure 5 and Figure S13, Table S8). This pathway schematic describes the combined alterations observed in multiple pathways, including steroid hormone biosynthesis, sphingolipid, glycerophospholipid, fatty acid, glycerolipid, amino acid and TCA cycle metabolism.

### 4.1. Dysregulation of Lipid and Fatty Acid Metabolism

Dysregulated sphingolipid metabolism is a central theme in many cancers, including ovarian cancer [25]. Although sphingolipids make up only a small fraction of the total lipid content, they control many fundamental cellular processes such as cell proliferation, differentiation, and apoptosis, thus playing a key role in cancer pathogenesis. Ceramides are powerful tumor suppressors and are central to sphingolipid metabolism. They can be formed by de novo synthesis, or by hydrolysis of sphingomyelins [35]. In agreement with previous studies [27], a decreased abundance of sphingomyelins and an apparent conversion to their hydrolysis products, ceramides, was observed in serum as well as in the tumor region of the TKO tissue, confirming the essential role of sphingolipid metabolism in HGSC (Figure S5).

In addition to sphingolipids, alterations in phospholipids, in particular LPC and PC, have been regularly documented in ovarian cancer studies [10]. LPC are the degradation product of PC and can be converted back to PC by lysophosphatidylcholine acyltransferases (LPCAT) in the presence of acyl-CoA enzymes. LPC, by binding and activating specific G protein-coupled receptors, regulate inflammatory response, oxidative stress, and cell proliferation [36], and, hence, are critical for cancer development. However, conflicting results have been reported on the changes in LPC/PC abundances associated with ovarian cancer. Some studies have reported increased LPC and decreased PC levels [37], whereas others have reported the opposite trend [38]. Although, our data suggests that most LPC species were lower in abundance in HGSC and most PCs were more abundant compared to controls, we also observed some lipid species following the opposite trend. PC species with odd fatty acid side chains such as PC(35:1) and PC(37:5), as well as some C36 and C38 PC species, were lower in TKO mice sera (Table S6). Similarly, MSI results showed an increase in PC(39:6) and a decrease in PC(20:4) in HGSC tumor regions (Figure 4 and Figure S5). Note that only two PC were putatively annotated in MSI experiments, which may have been caused by suppression of PC during (-)MALDI caused by the more acidic PE [39]. Among all annotated LPC species, only LPC(24:2) showed an overall increasing trend in TKO serum samples. Additionally, time-resolved analysis of LPC abundances in serum revealed an increasing temporal trend between 50 and 80 %lifetime, followed by a decreasing trend from 81–100 %lifetime (Figure 1), suggesting LPC levels are lower in metastatic cancer compared to early stages. The overall decrease in LPC levels observed in the HGSC (late stage) regions of the TKO tissues in MSI experiments supports the findings of the serum studies. The results for early vs. metastatic ovarian cancer were in line with observations by Ke et al., showing increased LPC levels in localized cancer compared to metastatic cancer in 448 plasma samples from OC patients [40]. Thus, it is possible that alterations in LPC/PC levels are specific to certain fatty acid acyl chain composition, or that literature inconsistencies arise from differences in the time or disease stages at which the samples are collected. Studies also suggest that such discrepancies are likely due to the complex enzymatic reactions involved in LPC/PC metabolism [36]. Nevertheless, our results indicated a time-dependent correlation of glycerophospholipid metabolism with HGSC development.

FA, as the building blocks of most lipid species, are essential for a range of cellular processes including membrane synthesis, energy storage and cell signaling. Increased fatty acids demand, either by de novo synthesis or by exogenous uptake, is essential to sustain the rapidly proliferating energy demands of cancer cells [41]. Additionally, the varying number of carbons and degrees of saturation impact the biophysical and chemical properties of FA [42]. In our lipidomics study, FA showed a variable trend depending on carbon chain length (Figure 1). The decreasing temporal trend for FA with less than or equal to 20 carbons could be attributed to increased mitochondrial FA β-oxidation for energy production. Conversely, we observed accumulation of fatty acids with more than 22 carbon chain length in TKO mice. Only FA(28:0) was lower in TKO animals compared to the controls. FA with carbon chain > 22 are referred to as very-long chain FA (VLCFA) and are metabolized in the peroxisome rather than in the mitochondria. Peroxisomes are oxidative organelles that are involved in fatty acid β-oxidation, ether-phospholipid synthesis, bile acid synthesis, and reactive oxygen species (ROS) homeostasis [43]. Low expression of ABCD1, the gene associated with the transport of VLCFA to the peroxisome for β-oxidation, has been linked with overall poor survival of ovarian cancer patients [27]. In addition to the VLCFA dysregulation, we observed reduced levels of ether phospholipids in TKO mice serum, as well as in the TKO tissue through MSI (Figure 1 and Figure S5). As noted earlier, ether phospholipids are synthesized in the peroxisomes and are involved in terminating ROS-induced lipid peroxidation [44]. Thus, dysregulated ether lipid and VLCFA metabolism suggests that peroxisomal dysfunction plays a critical role in ovarian cancer pathogenesis. Our results are in line with previous lipidomic ovarian cancer studies [10,27].

### 4.2. Progesterone Metabolism and Signaling in HGSC Development

An intriguing finding of this study is the potential role of progesterone metabolism in HGSC onset and development. We observed increased 20 α-OHP levels in TKO mice compared to controls in the early disease stages (37–80 %lifetime; log_2_ fold change TKO/Ctrl 0.99) and a decreasing trend in the advanced stages (85–100 %lifetime; log_2_ fold change −0.43) (Figure 1 and Figure S8). This metabolite, 20 α-OHP, is formed through the reduction in progesterone by 20-alpha-hydroxysteroid dehydrogenase (20 HSD), which is encoded by the AKR1C1 gene [45]. Aberrant expression of AKR1C1 has been reported for ovarian cancer tissues [46] and breast cancer cells [47], and studies have shown the crucial role of AKR1C1 in progesterone signaling [48]. Strikingly, our recent study of a mouse model of HGSC demonstrated that inhibition of ovarian progesterone signaling reduces ovarian cancer risk, highlighting the crucial role of progesterone signaling in ovarian cancer development [49]. Although progesterone itself was not well detected by our UHPLC-MS method, increased 20-OHP levels in the early disease stage may be indicative of increased progesterone metabolism signaling. Lower 20 α-OHP levels in advanced stage HGSC suggests decreased progesterone metabolism and progesterone signaling during tumor metastasis, which is consistent with our previous findings [49]. These are interesting findings that warrant further validation with targeted UHPLC-MS/MS approaches of these steroid pathways.

### 4.3. Inflammatory Metabolites Involved in HGSC Development

Another interesting finding was regarding the role of inflammatory mediators in HGSC tumor onset and progression. Among the annotated fatty acid chain composition of glycerophospholipids, glycerolipids, and sphingolipids, the linoleic acid (C18:2), arachidonic acid (C20:4), and docosahexaenoic acid (DHA, C22:6) fatty acid chains were frequently observed in our dataset (Table 2 and Table 3 and Table S6). Additionally, we observed increases in FA(20:4), FA(22:6) and FA(18:2) in the HGSC region of TKO tissue (Figure S5). Arachidonic acid, which is synthesized from linoleic acid, is a powerful inflammatory mediator and a substrate for other lipid signaling molecules such as eicosanoids, that are involved in inflammatory processes and tumorigenesis [50]. Inflammation is an established risk factor for ovarian cancer [51]. In fact, one reported strategy for reducing ovarian cancer risk is to reduce ovulation-induced inflammation with oral contraceptives [52]. Aspirin and other nonsteroidal anti-inflammatory drugs (NSAIDs) have also been reported as helping reduce ovarian cancer risk [53]. A recent study of wounded ovarian surface in a mouse model suggested inflammation as a potential mechanism for ovarian cancer development [54]. Our results suggest linoleic acid and arachidonic acid pathway-induced inflammation as a potential promotor (or product of) ovarian carcinogenesis. Further examination of the role of these specific metabolic pathways, and hence inflammation, will provide crucial information about the events associated with ovarian cancer initiation and progression.

### 4.4. Dysregulation of Biosynthetic Metabolism in HGSC Development and Progression

Our HILIC data revealed metabolites from arginine biosynthesis, TCA cycle, glycine, serine and threonine metabolism and tryptophan metabolism as essential associations with HGSC onset and progression (Figure 2). Alterations in the TCA cycle are a well-established signature of cancer metabolism, as it is crucial to central carbon metabolism and hence energy production and oxidative stress [55]. Besides the importance of TCA cycle intermediates in cancer metabolism, there is a growing consensus on the significance of amino acids in energy regulation and redox balance. In addition, studies have indicated that amino acid derivatives such as kynurenine, and arginine-derived polyamines are involved in immune responses associated with carcinogenesis [56]. Here, the reduced levels of amino acids and TCA cycle metabolites are consistent with findings of previous ovarian cancer studies [13]. Increased tryptophan degradation is one of the most frequently reported mechanisms of ovarian cancer. Studies suggest that increased tryptophan degradation by indoleamine-(2,3)-dioxygenase (IDO) leads to enhanced kynurenine production, which, in turn, triggers an immunosuppressive response [56]. Although our panel did not include kynurenine, the reduced tryptophan levels in TKO animals suggest that a dampened immune response could play a key role in HGSC onset and development. Furthermore, our results revealed the glycine, serine, and threonine pathway as another potential pathway of interest. Three metabolites for this pathway, serine, threonine, and 2-oxobutanoate were annotated as discriminatory species. The glycine, serine, and threonine pathway is the source for one-carbon metabolism and, consequently, regulates nucleotide synthesis as well as ATP and NADPH generation [57]. Serine is also a precursor for sphingolipid and glycerophospholipid synthesis. Additionally, increased N-acetylglutamine levels in TKO mice can be attributed to the increased production of acetyl-CoA for lipogenesis [15], TCA cycle, and cholesterol metabolism. Thus, when examined holistically, alterations observed in the polar metabolome and the lipidome of TKO mice reveal that systems level metabolic reprogramming collectively supports HGSC development and progression.

## 5. Conclusions

In conclusion, we present the first time- and space-resolved metabolomics/lipidomics study of HGSC development and progression in a mouse model faithfully representing human high-grade serous ovarian cancer, the most common and deadliest ovarian cancer type. Time-resolved analysis of animal serum revealed that alterations in the lipidome and metabolome start to occur with tumor onset, and that the observed changes are amplified as the disease progresses. Metabolites and lipids including ceramides, sphingomyelins, ether phospholipids, lysoglycerophospholipids, 20 α-hydroxyprogesterone, amino acids, and TCA cycle species showed altered profiles, even at the early disease stages, emphasizing their role in tumor onset. These alterations are indicative of disturbed mitochondrial and peroxisomal function, energy metabolism and redox homeostasis. Additionally, spatially resolved lipidomics experiments directly on reproductive tissues enabled visualizing the alterations and tissue heterogeneity within the tumor, which were more pronounced than in serum. Results showed the accumulation of different lipids in different sub-regions of the tumor, confirming that the observed serum lipid alterations are in fact a signature of HGSC. The spatial lipidomic experiments, however, were limited by the metabolite coverage. In this study, only ~1000 features were detected in the spatial lipidomics experiments, and ~150 of which were putatively annotated with FDR < 10%. The compound coverage of MSI experiments is mainly dependent on the ionization techniques applied (MALDI in this study), and specifically for MALDI MSI experiments, selection of the matrix and matrix deposition conditions usually lead to different compound coverage. Moreover, the TKO tissue sections in this study were collected when advanced tumors (HGSC) had developed; the spatial distribution and alterations of the lipids remains unknown in the benign tumors at earlier stages. Therefore, it would be desirable to image tissues collected from different animals at different OC stages, using various MSI approaches. Nonetheless, these experiments offered a first in-depth view of the molecular signature of HGSC, and future work will involve visualizing the tumor tissue of early stage HGSC. Additionally, although the TKO animals provide a simpler, better controlled, and a faithful representation of human HGSC, the reported findings need to be validated in a human cohort and the overlap between the metabolic dysregulation observed in TKO animals and humans needs to be explored. Overall, our study offers a comprehensive understanding of the metabolic changes associated with the initiation, development, and progression of HGSC, thereby providing avenues for the discovery of early stage HGSC biomarkers.

## Figures and Tables

**Table 1:** Metabolite annotations for the 14 selected features in the HILIC dataset. Proposed metabolite annotation, experimental monoisotopic *m*/*z* value, elemental formula, chromatographic retention time (min), mass error (ppm), main adduct type detected, abundance log-transformed fold changes, and metabolite annotation confidence level are shown. Fold changes were calculated as the base 2 logarithm of the average abundance ratios between TKO and TKO controls in the 37–60 %lifetime group. Positive values indicate higher levels in TKO serum samples and negative values indicate lower levels in TKO serum samples compared to TKO controls. The confidence level for metabolite annotation was assigned as (1) exact mass, isotopic pattern, retention time, and MS/MS spectrum of a chemical standard matched to the feature. (2) exact mass, isotopic pattern, retention time, and MS/MS spectrum matched to an in-house spectral database or literature spectra (3) putative ID assignment based only on elemental formula match. (4) unknown compound. Compound classes are provided for features with low annotation confidence levels.

ID	Elemental Formula	Proposed Annotation	Adduct Type	Experimental *m*/*z*	Retention Time (min)	Mass Error (ppm)	Log_2_ Fold Change TKO/Ctrl	*q*-Value	Annotation Confidence Level
78	C_4_H_6_O_3_	oxobutanoic acid	[M−H]^−^	101.0245	5.11	0.59	−0.36	0.03	2
92	C_3_H_7_NO_3_	serine	[M−H]^−^	104.0354	8.71	0.58	−0.23	0.03	2
159	C_4_H_9_NO_3_	threonine	[M−H]^−^	118.0509	8.35	−0.25	−0.23	0.03	2
242	C_5_H_12_N_2_O_2_	ornithine	[M−H]^−^	131.0827	8.85	0.77	−0.17	0.03	2
251	C_4_H_6_O_5_	malic acid	[M−H]^−^	133.0143	7.10	0.70	−0.47	0.03	2
345	C_5_H_6_O_5_	oxoglutaric acid	[M−H]^−^	145.0143	5.13	0.18	−0.28	0.04	2
539	C_6_H_13_N_3_O_3_	citrulline	[M−H]^−^	174.0883	8.85	−0.28	−0.17	0.03	2
635	C_7_H_12_N_2_O_4_	N-acetylglutamine	[M−H]^−^	187.0724	4.90	−0.09	0.26	0.04	2
698	C_6_H_8_O_7_	citric acid/isocitric acid	[M−H]^−^	191.0197	6.67	0.02	−0.23	0.03	2
807	C_11_H_12_N_2_O_2_	tryptophan	[M−H]^−^	203.0824	6.67	−0.76	−0.31	0.03	2
1742	C_24_H_38_O_2_	Bile acids and derivatives	[M−H]^−^	357.2796	1.17	−0.69	0.54	0.03	2
1766	C_20_H_30_O_6_	hydroxy-12-oxo-eicosatrienedioic acid or trihydroxy-12-keto-eicosatetraenoic acid	[M−H]^−^	365.1966	1.19	−0.94	−0.44	0.02	2
1780	C_24_H_38_O_3_	Bile acids and derivatives	[M−H]^−^	373.2744	1.20	−1.19	0.49	0.05	2
1807	C_20_H_30_O_7_	tetrahydroxy-12-oxo-tetraenoic acid	[M−H]^−^	381.1917	1.10	−0.51	−0.18	0.04	2

**Table 2:** Optimized discriminant lipid panel for early stage HGSC. Variables were selected using a genetic algorithm (GA) for optimum discrimination of 37–60 %lifetime TKO and TKO control samples. Proposed metabolite annotation, experimental monoisotopic *m*/*z* value, elemental formula, chromatographic retention time (min), mass error (ppm), main adduct type detected, abundance log-transformed fold changes, and metabolite annotation confidence level are shown. Fold changes were calculated as the base 2 logarithm of the average abundance ratios between TKO and TKO controls in the 37–60 %lifetime group. Positive values indicate higher levels in TKO serum samples and negative values indicate lower levels in TKO serum samples compared to TKO controls. The confidence level for metabolite annotation was assigned as (1) exact mass, isotopic pattern, retention time, and MS/MS spectrum of a chemical standard matched to the feature. (2) exact mass, isotopic pattern, retention time, and MS/MS spectrum matched to an in-house spectral database or literature spectra (3) putative ID assignment based only on elemental formula match. (4) unknown compound. Abbreviations: DG: Diacylglycerols, TG: Triacylglycerols, FA: Fatty acids, HexCer: Hexosylceramides, LPC: Lysophosphatidylcholines, LPE: Lysophosphatidylethanolamines, PC: Phosphatidylcholines, PC-O: Ether phosphatidylcholines, PE: Phosphatidylethanolamines, PE-O: Ether phosphatidylethanolamines, PI: Phosphatidylinositols, PS: Phosphatidylserines, SiE: Stigmasterol ester, Cer: Ceramides, and SM: Sphingomyelins.

Feature ID	Annotation	Adduct Type	Experimental *m*/*z*	Elemental Formula	Retention Time (min)	Log_2_ Fold Change TKO/Ctrl	Mass Error (ppm)	Confidence Level
3968	20alpha-hydroxyprogesterone	[M+H]^+^	317.2476	C_21_H_32_O_2_	1.35	0.90	0.32	2
2209	Cer(d41:1)	[M+CH_3_COOH−H]^−^	694.6365	C_41_H_81_NO_3_	7.36	0.12	1.40	2
96	citrulline	[M−H]^−^	174.0885	C_6_H_13_N_3_O_3_	1.02	−0.22	0.67	2
473	FA(16:1)	[M−H]^−^	253.2174	C_16_H_30_O_2_	2.28	−0.01	0.49	2
1253	FA(28:0)	[M−H]^−^	423.4209	C_28_H_56_O_2_	6.89	−0.36	0.39	2
4628	LPC(0:0_18:2)	[M+H]^+^	520.3401	C_26_H_50_NO_7_P	1.77	−0.25	0.54	2
4738	LPC(20:4)	[M+H]^+^	544.3402	C_28_H_50_NO_7_P	1.81	−0.10	0.86	2
5068	LPC(24:2)	[M+H]^+^	604.4336	C_32_H_62_NO_7_P	2.77	0.09	−0.17	2
1257	LPE(O-15:0)	[M−H]^−^	424.2851	C_20_H_44_NO_6_P	2.28	−0.07	4.07	2
2668	PC(17:0_18:2)	[M+H_2_CO_2_−H]^−^	816.5782	C_43_H_82_NO_8_P	4.56	0.01	2.69	2
7098	PC(41:6)	[M+H]^+^	848.6162	C_49_H_86_NO_8_P	5.22	0.05	−0.22	2
5785	PC(O-16:0_18:2)	[M+H]^+^	744.5903	C_42_H_82_NO_7_P	5.34	−0.18	0.19	2
6406	PC(O-38:2)	[M+H]^+^	800.6534	C_46_H_90_NO_7_P	6.55	−0.23	0.72	2
2297	PE(34:0)	[M−H]^−^	718.5404	C_39_H_78_NO_8_P	6.18	0.07	1.71	2
2475	PE(36:1)	[M−H]^−^	772.5873	C_43_H_84_NO_8_P	6.89	0.02	1.45	2
2406	PE(17:0_20:4)	[M−H]^−^	752.5243	C_42_H_76_NO_8_P	5.00	−0.16	1.01	2
5982	PE(18:0_20:4)	[M+H]^+^	768.5545	C_43_H_78_NO_8_P	5.24	−0.13	0.89	2
7279	PI(18:0_18:2)	[M+H]^+^	863.5644	C_45_H_83_O_13_P	4.56	−0.17	0.03	2
2543	SM(d36:1)	[M+H]^+^	731.6069	C_41_H_83_N_2_O_6_P	4.87	−0.16	0.95	2
2733	SM(d40:2)	[M+H_2_CO_2_−H]^−^	829.6458	C_45_H_89_N_2_O_6_P	6.20	−0.13	2.21	2
7627	TG(53:2)	[M+NH_4_]^+^	890.818	C_56_H_104_O_6_	9.08	−0.45	0.91	2
8173	TG(18:1_18:1_20:4) and TG(16:0_18:1_22:5) and TG(18:1_18:2_20:3)	[M+NH_4_]^+^	924.8022	C_59_H_102_O_6_	8.30	−0.19	0.71	2

**Table 3:** Selection of lipid ratio abundance biomarkers based on their significant differences between TKO and TKO control samples. The selected ratios were validated with a 10-fold cross-validated logistic regression model. The area under the curve (AUC), sensitivity, and specificity are shown for (a) all TKO and TKO controls samples in the 37–100 %lifetime group (b) TKO and TKO controls in 37–60 %lifetime group. CI: confidence interval.

**(a) 37–100 %lifetime TKO vs. TKO Ctrl**				
**Lipid Ratio Biomarker**	***p*-Value**	**AUC (95% CI)**	**Sensitivity (95% CI)**	**Specificity (95% CI)**
Cer(d34:1)/PE(O-18:1_18:2)	3.27 × 10^−24^	0.830 (0.785~0.876)	0.744 (0.744~0.818)	0.774 (0.710~0.830)
Cer(d34:1)/PE(O-20:0_18:2)	3.27 × 10^−23^	0.828 (0.781~0.875)	0.774 (0.774~0.845)	0.768 (0.704~0.833
Cer(d34:1)/PE(O-20:1_22:6)	4.78 × 10^−23^	0.819 (0.771~0.867)	0.737 (0.737~0.812)	0.780 (0.717~0.844)
Cer(d34:1)/PE(O-40:5)	2.92 × 10^−23^	0.819 (0.771~0.868)	0.722 (0.722~0.798)	0.793 (0.731~0.855)
Cer(d34:1)/PE(O-20:1_22:4)	2.92 × 10^−23^	0.818 (0.770~0.865)	0.752 (0.752~0.825)	0.720 (0.651~0.788)
**(b) 37–60 %lifetime TKO vs. TKO Ctrl**				
**Lipid Ratio Biomarker**	***p*-Value**	**AUC (95% CI)**	**Sensitivity (95% CI)**	**Specificity (95% CI)**
Cer(d34:1)/PE(O-18:1_18:2)	2.01 × 10^−7^	0.763 (0.673~0.854)	0.745 (0.745~0.869)	0.710 (0.597~0.823)
Cer(d34:1)/PE(O-20:0_18:2)	1.25 × 10^−6^	0.756 (0.665~0.848)	0.787 (0.787~0.904)	0.677 (0.561~0.794)
Cer(d34:1)/PE(O-20:1_22:6)	9.13 × 10^−6^	0.729 (0.630~0.827)	0.723 (0.723~0.851)	0.677 (0.561~0.794
Cer(d34:1)/PE(O-40:5)	4.70 × 10^−7^	0.734 (0.638~0.831)	0.660 (0.660~0.795)	0.758 (0.651~0.865)
Cer(d34:1)/PE(O-20:1_22:4)	1.53 × 10^−5^	0.718 (0.621~0.814)	0.681 (0.681~0.814)	0.645 (0.526~0.764)

## Data Availability

Data generated in this study are available through the NIH Metabolomics Workbench (http://www.metabolomicsworkbench.org/ with project ID PR001309 (for LC-MS data the study ID is ST002067 [http://dx.doi.org/10.21228/M8HH60]; MSI data study ID is ST002083 [http://dx.doi.org/10.21228/M8HH60]).

## Supplementary Materials

The following supporting information can be downloaded at: https://www.mdpi.com/article/10.3390/cancers14092262/s1, Figure S1. Alignment of time-course data based on %lifetime. Figure S2. Abundance fold change as a function of %lifetime for (a) total LPC and (b) total ceramides at various HGSC stages. Figure S3. Representative chromatographic data. Figure S4. Unsupervised and supervised multivariate analysis of RP UHPLC-MS data using only the abundances of statistically-significant compounds as input. Figure S5. Alterations in the serum lipidome at various HGSC stages. Figure S6. Heatmaps showing average abundance changes between TKO and TKO control animals for individual lipids. Figure S7. Time-resolved serum lipidomics. Figure S8. 20α-hydroxyprogesterone (20α-OHP) levels during the tumor progression in TKO vs TKO control samples. Figure S9. Unsupervised and supervised multivariate analysis of statistically-significant (-) HILIC UHPLC-MS compounds. Figure S10. Supervised multivariate analysis of (-) HILIC early stage data. Figure S11. Supervised oPLS-DA multivariate analysis of optimized feature sets. Figure S12. Comparison of serum lipid ratios for various %lifetimes in TKO and TKO control animals. Figure S13. Pathway analysis of HILIC data. Figure S14. Unsupervised Principal Component Analysis (PCA). Table S1. Composition of internal standards used in UHPLC-MS. Table S2. Chromatographic gradients for RP and HILIC methods. Table S3. MS parameters. Table S4. Serial serum collection information. Table S5. Age of TKO and TKO control mice with their corresponding %lifetime values. Table S6. Annotated compounds with statistically significant differential abundances in the RP dataset. Table S7. Metabolite annotations for the 46 selected features in the HILIC dataset. Table S8. Pathway analysis results obtained from Lipid Pathway Enrichment Analysis (LIPEA) of RP data. Reference in supplementary [58,59,60,61,62].
