# Supplementary material for: Space- and Time-Resolved Metabolomics of a High-Grade Serous Ovarian Cancer Mouse Model
Source: Cancers (Basel). 2022 Apr 30;14(9):2262. doi: 10.3390/cancers14092262 (PMC9104348; doi:10.3390/cancers14092262)
Supplement: Supplementary file 1 [file cancers-14-02262-s001.zip › cancers-1680945-supplementary.pdf]

Supplementary Materials Space- and Time-resolved Metabolomics of a High-Grade Serous Ovarian Cancer Mouse Model.

Samyukta Sah, Xin Ma, Andro Botros, David A. Gaul, Sylvia R. Yun, Eun Young Park, Olga Kim, Samuel G. Moore, Jaeyeon Kim, Facundo M. Fernández

UHPLC-MS Metabolite Extraction Protocol

Reverse phase (RP) ultra-high-performance liquid chromatography – mass spectrometry (UHPLC-MS) and hydrophilic interaction liquid chromatography (HILIC) UHPLC-MS analysis were performed to obtain a deeper coverage of the metabolome. Serum samples were thawed on ice, followed by metabolite extraction of both non-polar (lipid) and polar metabolites using two different sample preparation protocols. The extraction solvent for RP UHPLC-MS was prepared by adding the isotopically labeled lipid standard mixture (Table S1) to 2-propanol in a 1:60 ratio. Likewise, for HILIC UHPLC-MS, the extraction solvent was prepared by mixing an isotopically labeled mixture of polar metabolites (Table S1) and methanol in a 1:60 ratio. These cold extraction solvents were added to serum samples in a 3:1 ratio to precipitate proteins. Following this step, samples were vortex-mixed for 30 s and centrifuged at 13,000 rpm for 7 min. The resulting supernatant was transferred to LC vials with snap-on caps and stored at -80 °C until UHPLC-MS analysis, which was performed within a week. A blank sample, prepared with LC-MS grade water underwent the same preparation process as the serum samples. A pooled quality control (QC) was prepared by mixing 5-10 μL aliquots of the extract of each serum sample. This pooled sample was analyzed every 10 runs to monitor LC-MS instrument stability and later used to correct for any instrument drift. Samples were run in a randomized order on consecutive days.

UHPLC-MS Quality Control

Prior to multivariate data analysis, the quality of pooled QC runs was evaluated. Relative standard deviation (RSD) of the average intensities of all QC files were calculated with RawMeat (Vast Scientific) software. RSD <15% was obtained for each positive and negative ion mode RP UHPLC-MS datasets and the negative ion mode HILIC UHPLC-MS dataset. A tight clustering of the QC runs in the PCA score plots (Figure S14) was observed, indicating excellent reproducibility. Additionally, all TKO and QC samples across the batch were evaluated using peak areas extracted with Compound Discoverer v3.1 (ThermoFisher Scientific) for each detected internal standard. Although both negative and positive ion modes datasets were acquired for HILIC UHPLC-MS, only the negative ion mode dataset was used for further analysis. The positive mode HILIC UHPLC-MS dataset contained a large number of noisy features that made peak area integration impossible and was therefore removed from the analysis. The amount of serum available after all UHPLC-MS experiments were performed prevented positive ion mode HILIC experiments to be repeated.

UHPLC-MS/MS Experiments and Metabolite Annotation

Q Exactive HF MS/MS experiments were performed in a data-dependent acquisition (DDA) fashion using stepped normalized collision energies (NCE) of 10, 30 and 50 to fragment the precursor ions in the HCD cell. Orbitrap ID-X DDA experiments were performed using the Thermo Scientific AcquireX data acquisition workflow. Stepped NCE of 15, 30, 45 and a CID collision energy of 35 was used to fragment the precursor ions. For compounds that were missed in DDA experiments, MS/MS spectra were acquired in a targeted fashion using an inclusion list of the precursor ions. Stepped NCE of 15, 30, 45 and a CID collision energy of 40 was used for the targeted MS/MS experiments.

Metabolite annotation was attempted for all spectral features in the RP dataset, and for selected discriminant features in the HILIC dataset. The exact masses, retention times, and MS/MS spectra of all RP features were matched against a curated in-house spectral database. Features that did not have matches in our local MS/MS database were matched against databases such as Lipid Maps [58] and mzCloud [59]. For features in the HILIC dataset, elemental formulas were generated based on exact masses and isotopic pattern matching using Compound Discoverer v3.1. The generated elemental formulas and exact masses with a maximum mass error of 5 mDa were searched against metabolomic databases such as Metlin [60] and the human metabolome database [61] (HMDB) to assign tentative annotations. Product ion spectra obtained from MS/MS experiments were analyzed and matched against tandem MS databases including MassBank [62][1], Metlin and mzCloud to confirm metabolite annotations.

Tissue Preparation for MS Imaging Experiments

The freshly frozen tissues were embedded in an aqueous solution containing 1 % CMC and 5 % (by weight) gelatin. Tissue embedding was conducted in an isopentane-dry ice bath at -20 °C. A CryoStar NX70 Cryostat was used for cryosectioning.

The sectioning temperature was set to -20 °C and each slice was sectioned at a thickness of 10 µm. Sectioned tissue slices were transferred to Fisherbrand™ Superfrost™ Plus microscope slides for MALDI imaging MS experiments. Mounted tissue slices were sprayed with 5 mg mL^{-1} 1,5-DAN prior to MALDI MS. 1,5-DAN was dissolved in 65/20/15 (v/v/v) acetonitrile/methanol/chloroform and sprayed *via* an iMatrix matrix sprayer. The sprayer nozzle height was set to 60 mm, the speed of the nozzle movement was 200 mm s^{-1}. The inter-line distance was 1 mm. One µL of the matrix solution was sprayed onto an area of 1 cm^2 on average. The spray cycle was repeated 10 times to ensure complete and uniform matrix coverage on tissue sections.

FTICR Conditions for Imaging Experiments

The MALDI laser power was set to 25%, and the number of laser shots accumulated on each pixel was 300. The laser repetition frequency was 1000 Hz, and the laser beam focus size was set to minimum. The spatial resolution defined by the pixel size of the images was 50 µm × 50 µm. A typical image required ~40 hours of data acquisition, yielding files of 800 GB. The time domain data set size was set to 4,000,000, corresponding to a mass resolution of 410,000 at *m/z* 400, and the FID transient time was 0.4194 s. The mass spectrometer was calibrated externally with (+)ESI and (-)ESI CalMix solution and internally with FA(18:1) and PI(38:4) to ensure mass accuracy was better than 1 ppm on average

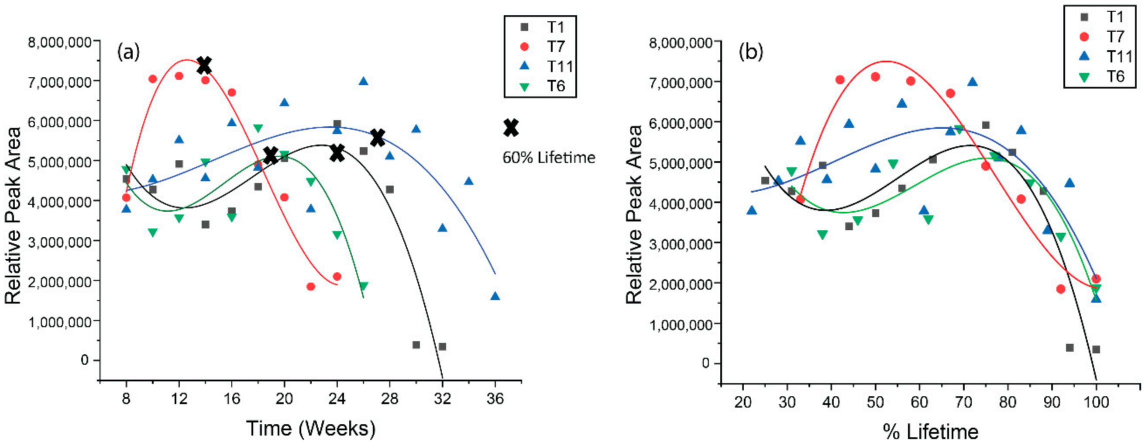

$$\text{Lifetime} = \frac{\textit{Age of mouse in months}}{\textit{Total life span of the mouse or age at last time point of blood collection}} \times 100$$

Figure S1. Alignment of time-course data based on %lifetime. (a) Time course for the abundance of LPC(20:3) in 4 different TKO mice labeled T1, T6, T7 and T11. The x-axis represents time in weeks and the y-axis shows the relative peak abundance of the lipid LPC(20:3). Time course data for each animal were fitted with a cubic polynomial function for better visualization. The 60 %lifetime is indicated by ✖ on the polynomial curve for each specific mouse, to emphasize the different lifespans observed. (b) Time course of LPC(20:3) abundance in TKO mice T1, T6, T7 and T11 after aligning the data with the %lifetime variable. The x-axis shows the %lifetime, calculated as the ratio of the age of mouse at a given serum sampling time point compared to the total lifespan of that specific mouse. The y-axis shows the relative peak abundance of the lipid LPC(20:3). .

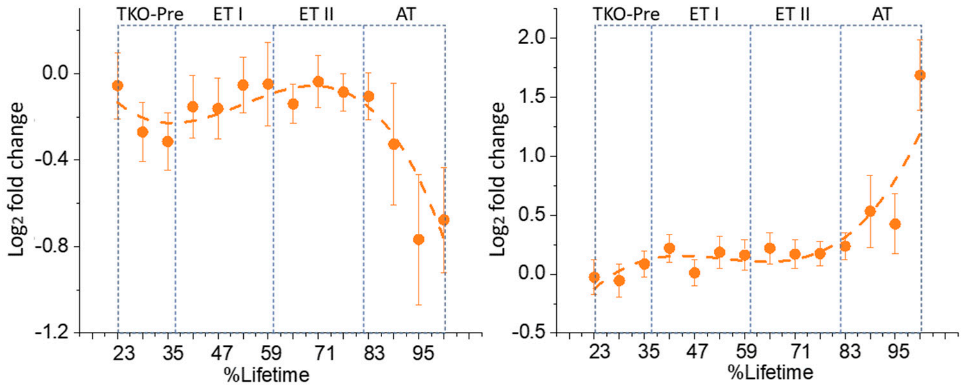

Figure S2. Abundance fold change as a function of %lifetime for (a) total LPC and (b) total ceramides at various HGSC stages. "TKO-Pre" at 20-36 %lifetime (TKO n=21; TKO ctrl n=36) corresponds to premalignant stages, "ET-I" at 37-60 %lifetime (TKO n=46; TKO ctrl n=62) is early-stage I and most likely corresponds to tumor onset stages, "ET-II" at 61-80 %lifetime, (TKO n=36; TKO ctrl n=46) is early stage II and corresponds to early-stage HGSC with no sign of metastasis, "AT" at 81-100 %lifetime (TKO n=46; TKO ctrl n=53) is advanced-stage. The total abundance for each lipid class was calculated by averaging the relative abundances of all statistically significant lipid species in that specific class. The x-axis shows %lifetime, calculated as the ratio of the age of mouse at a given serum sampling time point compared to the total lifespan of that specific mouse. The y-axis shows the fold change calculated as the base 2 logarithm of the lipid content ratios of TKO / TKO control samples. Positive values indicate higher serum levels in TKO animals, and negative values indicate lower serum levels in TKO animals compared to TKO controls. Error bars represent the standard error of the $\log_2$ fold change between TKO and control samples.

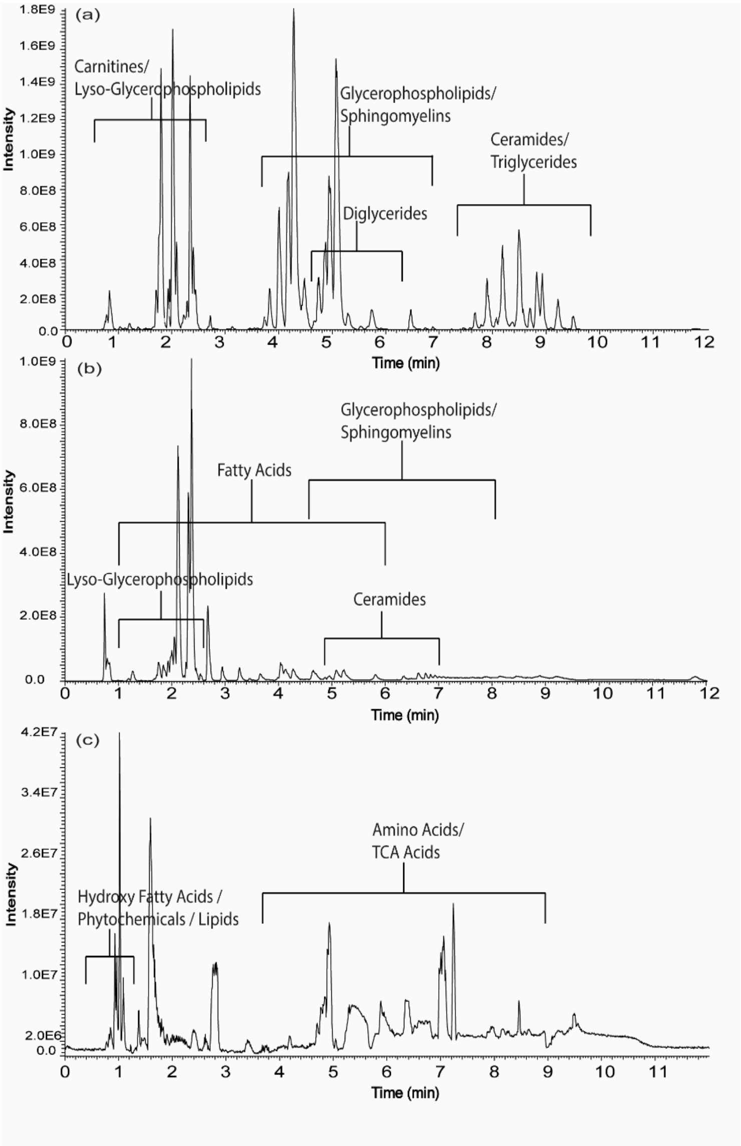

Figure S3. Representative chromatographic data. Base peak intensity chromatograms for pooled QC serum samples obtained with (a) (+) RP UHPLC-MS, (b) (-) RP UHPLC-MS and (c) (-) HILIC UHPLC-MS. The x-axis represents chromatographic retention time in minutes, and the y-axis shows the MS peak abundance. Lipid classes observed with RP experiments and polar metabolites observed with HILIC experiments are labeled based on their elution regions.

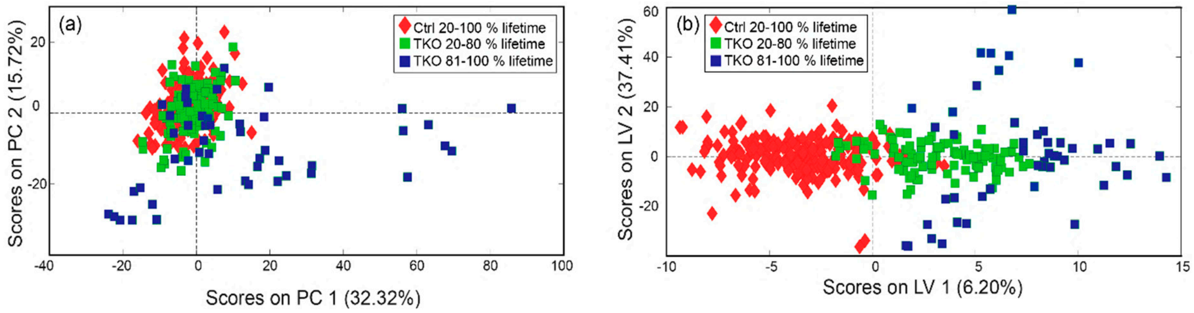

Figure S4. Unsupervised and supervised multivariate analysis of RP UHPLC-MS data using only the abundances of statistically-significant compounds as input. (a) PCA score plot showing clustering of TKO and TKO control samples using the combined set of statistically significant (+) and (-) RP UHPLC-MS features. Features were selected with Welch's t-test with a Benjamini-Hochberg correction. (b) oPLS-DA score plot for the same dataset. TKO samples at 20-80 %lifetime are represented with green squares, TKO samples at 81-100 %lifetime are shown as blue squares, and TKO control samples at 20-100 %lifetime are shown as red diamonds.

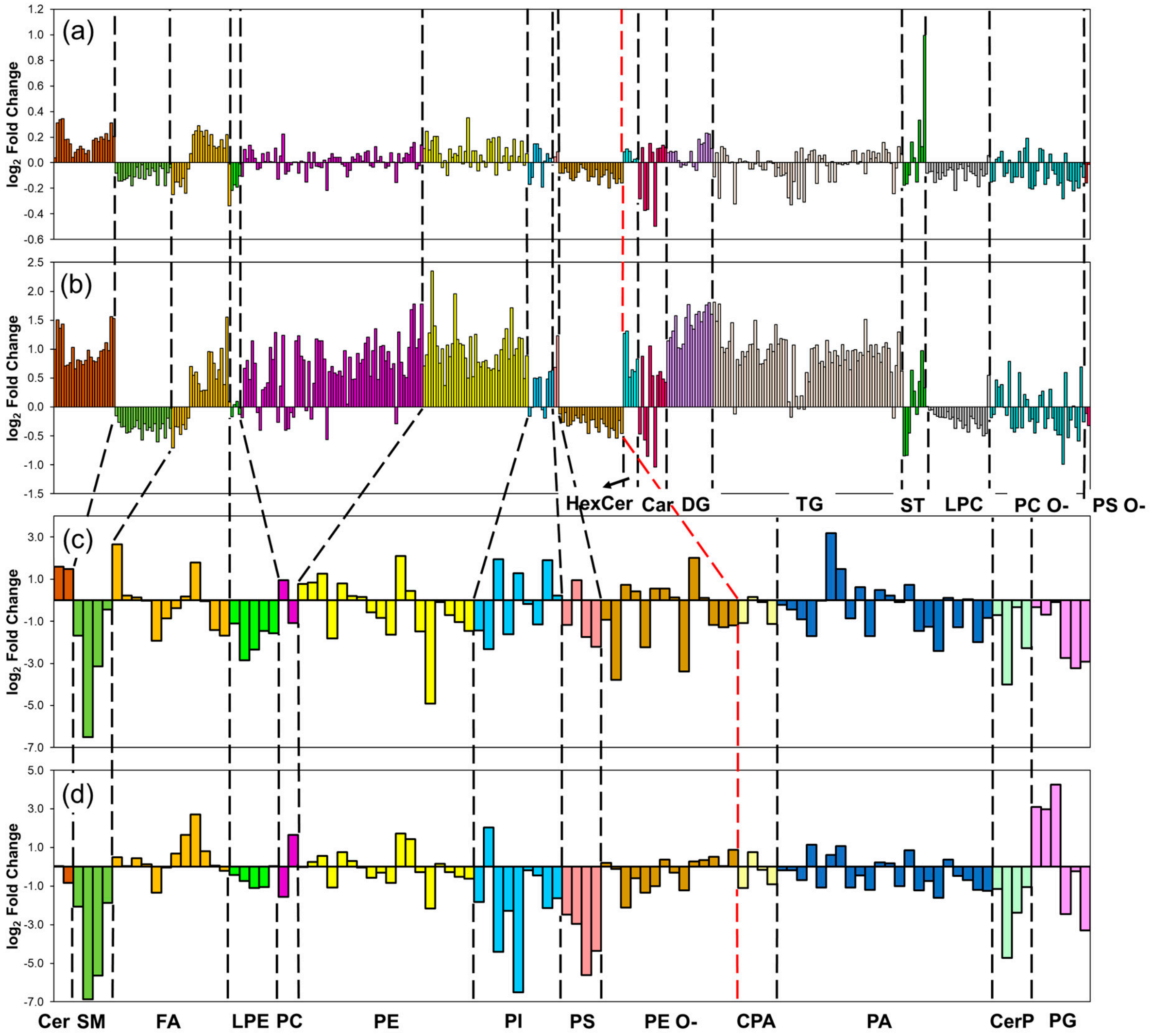

Figure S5. Alterations in the serum lipidome at various HGSC stages. Fold changes were calculated as the base 2 logarithm of the average lipid abundance ratios for (a) TKO *vs*. control UHPLC-MS serum abundances at 36–80 %lifetime, (b) TKO *vs*. control UHPLC-MS serum abundances at 81–100 %lifetime, (c) HGSC region of interest (ROI) in a TKO animal *vs*. healthy ovary tissue ROI in a TKO control animal imaged with FTICR MS, and (d) HGSC ROI in a TKO animal *vs*. healthy fallopian tube ROI in a TKO control animal, also imaged with FTICR MS. Positive values indicate higher levels in TKO samples. Negative values indicate lower levels in TKO samples compared to controls. Compounds listed to the left of the red dashed line were annotated in both serum (UHPLC-MS) and tissues (MSI). Cer: Ceramides, SM:

Sphingomyelins, FA: Fatty acids, LPE: Lysophosphatidylethanolamines, PC: Phosphatidylcholines, PE: Phosphatidylethanolamines, PI: Phosphatidylinositols, PS: Phosphatidylserines, PE O-: Ether phosphatidylethanolamines, HexCer: Hexosylceramides, Car: Carnitines, DG: Diacylglycerols, TG: Triacylglycerols, ST: Sterol lipids, LPC: Lysophosphatidylcholines, PC O-: Ether phosphatidylcholines, and PS O-: Ether phosphatidylserines, CPA: Cyclopropane fatty acids, PA: Phosphatidic acids, CerP: Ceramide phosphates, PG: Phosphatidylglycerols.

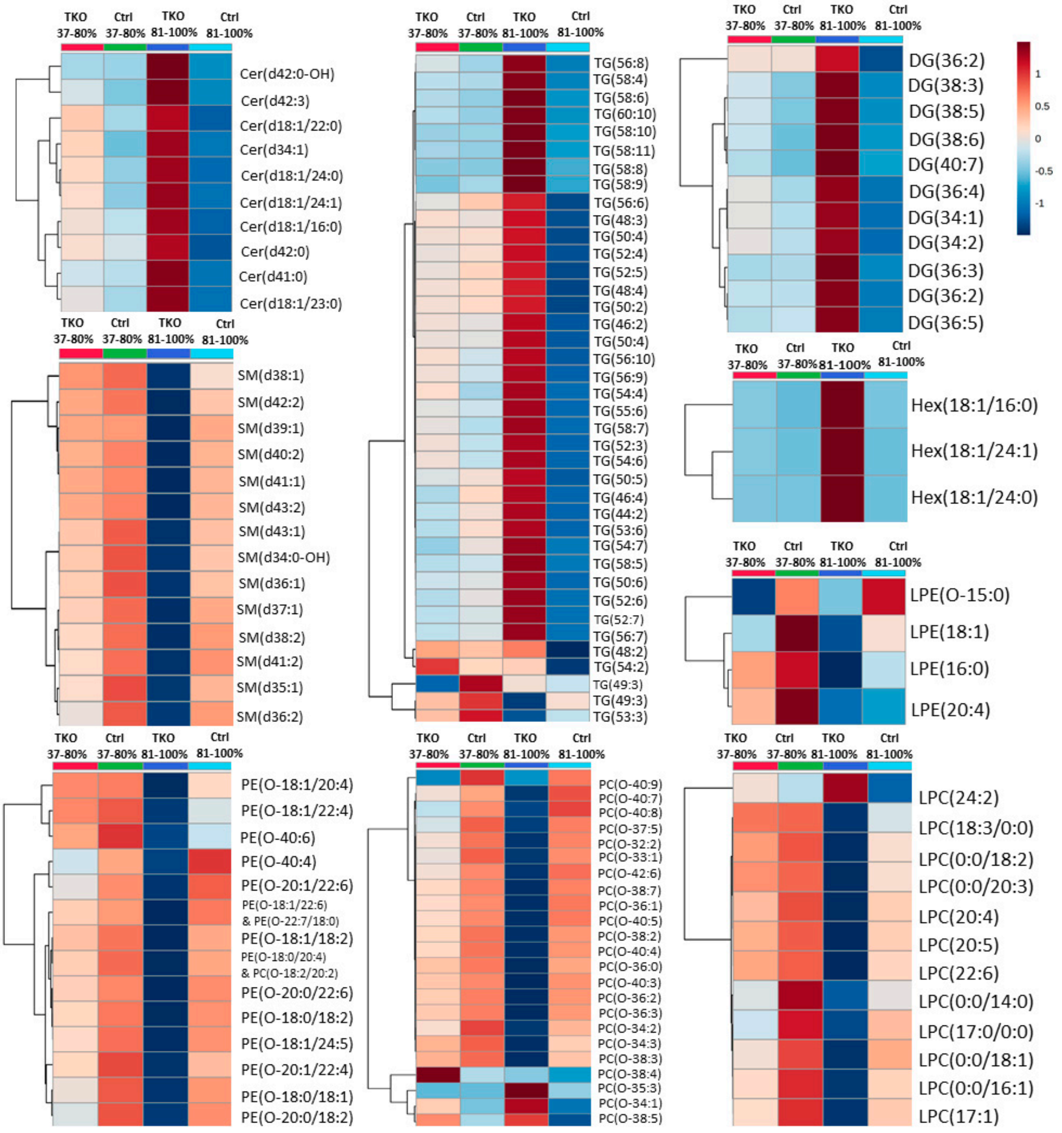

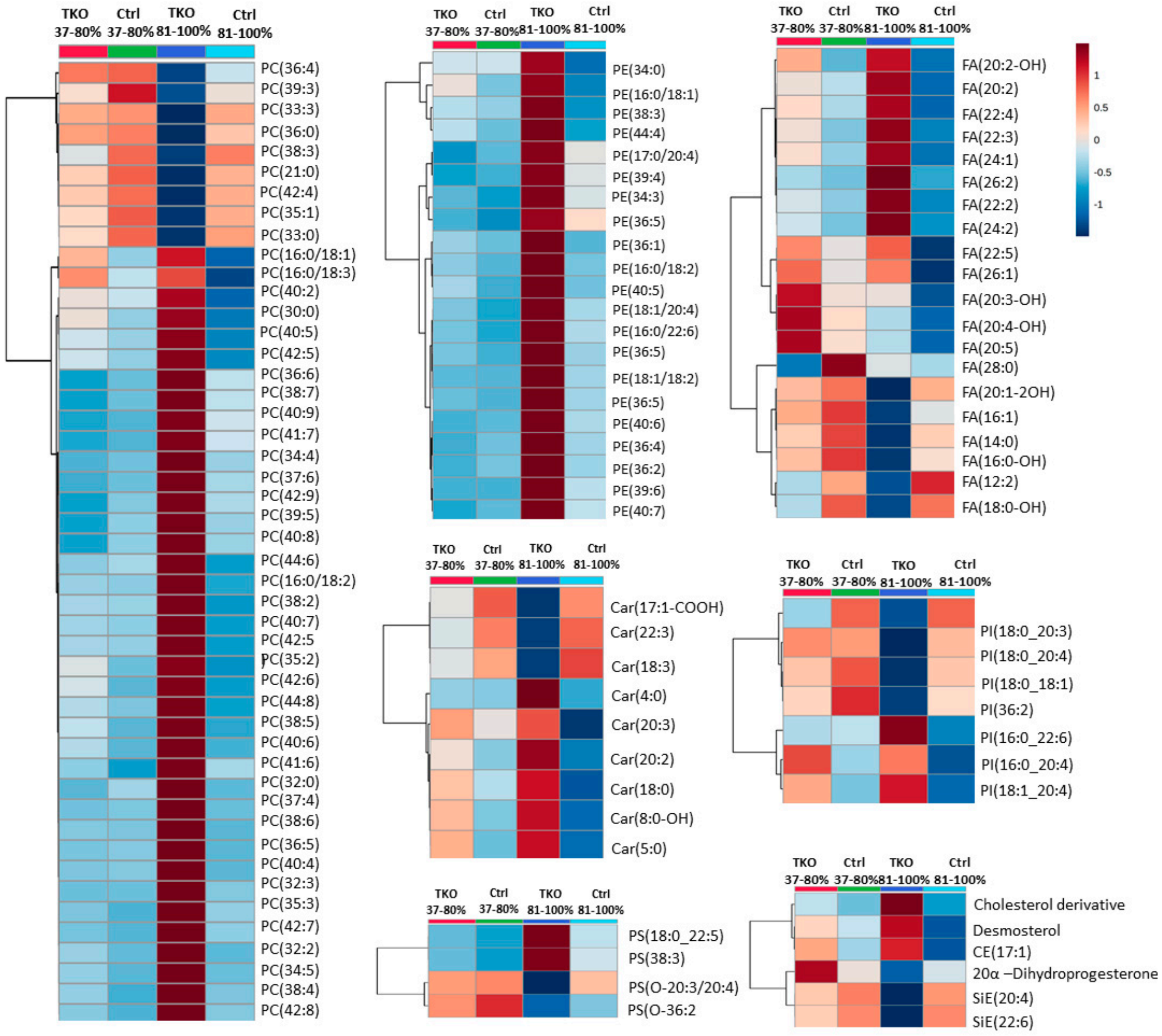

Figure S6. Heatmaps showing average abundance changes between TKO and TKO control animals for individual lipids. Only statistically significant lipids are shown. The color scale represents the Z-score, with red indicating higher mean abundance and blue indicating lower mean abundance. Pearson correlation was used for clustering. All compound abundances were autoscaled. In cases where isomers were resolved, only average abundances of one lipid isomer are shown. Abbreviations: Cer: ceramides, SM: Sphingomyelins, HC: Hexosylceramides, Car: Carnitines, DG: Diacylglycerols, TG: Triacylglycerols, FA: Fatty acids, ST: Sterol lipids, LPC: Lysophosphatidylcholines, LPE: Lysophosphatidylethanolamines, PE: Phosphatidylethanolamines, PC: Phosphatidylcholines, PI: Phosphatidylinositols, PS: Phosphatidylserines, PE O-: Ether phosphatidylethanolamines, PC O-: Ether phosphatidylcholines, and PS O-: Ether phosphatidylserines.

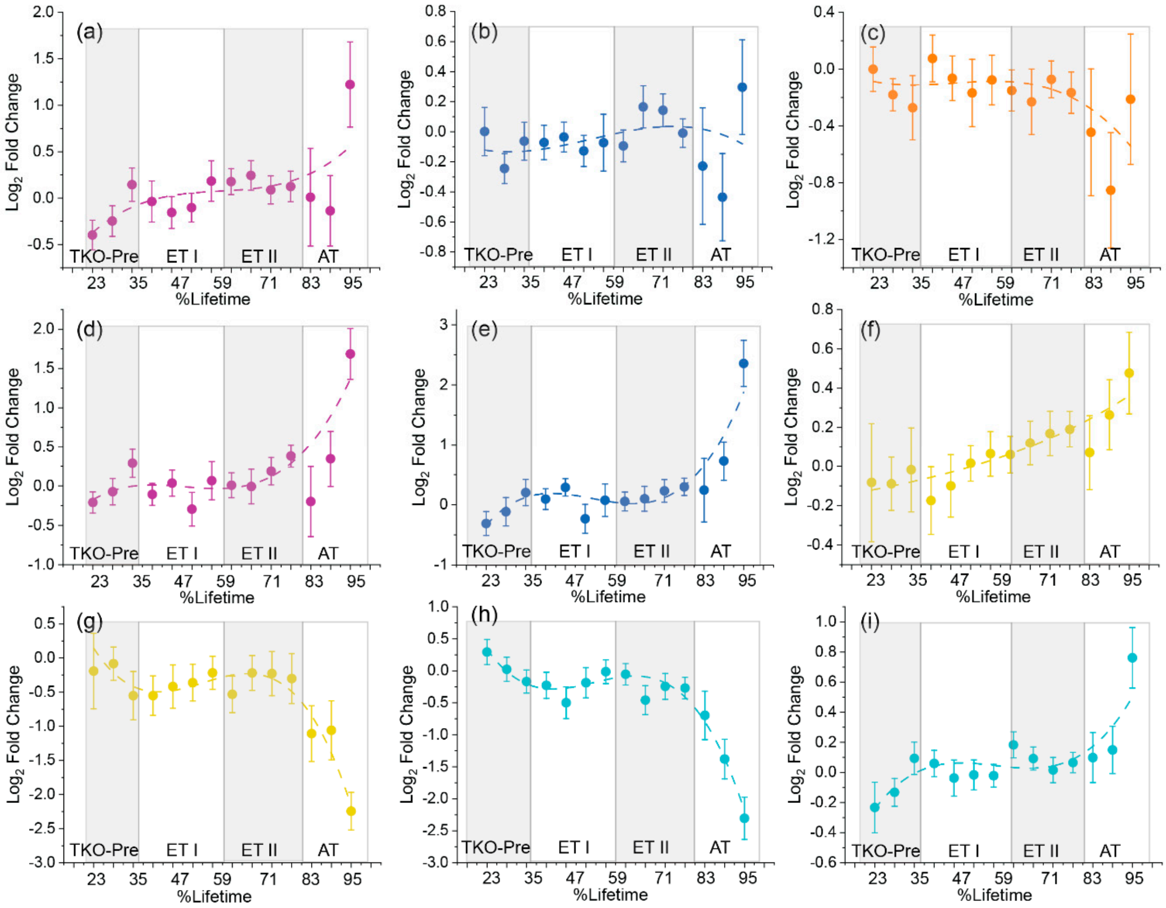

Figure S7. Time-resolved serum lipidomics. Abundance fold-changes in serum as a function of %lifetime for (a) Phosphatidylserines (PS) b) Phosphatidylinositols (PI) (c) Ether Phosphatidylserines (PS O-) (d) Triacyglycerols (TG) (e) Diacyglycerols (DG) (f) Carnitine (Car)(4:0), Car(5:0), Car(20:3), Car(20:2), Car(18:0), Car(8:0-OH) (g) Car(17:1-COOH), Car(22:3), Car(18:3) (h) Stigmasterols (i) Cholesterol derivatives. The total lipid abundance for each lipid class was calculated by averaging the relative peak abundances of all statistically significant lipid species in that specific class. The x-axis shows the %lifetime, calculated as the ratio of the age of mouse at a given serum sampling time point compared to the total lifespan of that specific mouse. The y-axis shows the fold change calculated as the base 2 logarithm of the lipid content ratios of TKO / TKO control samples. Positive values indicate higher serum levels in TKO animals, and negative values indicate lower serum levels in TKO animals compared to TKO controls. Error bars represent the standard error of the $\log_2$ fold change between TKO and control samples. HGSC stage estimates at different %lifetime are shown. TKO-pre corresponds to premalignant stages, ET-I is early-stage 1, ET-II is early-stage II and AT is advanced stage.

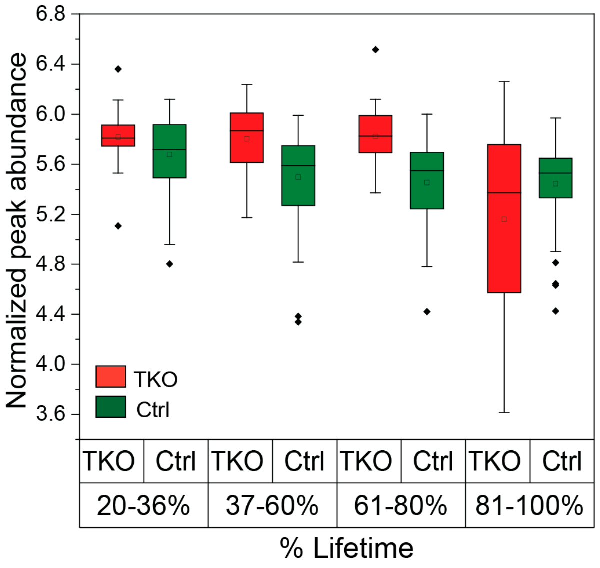

Figure S8. 20α-hydroxyprogesterone (20α-OHP) levels during the tumor progression in TKO vs TKO control samples. Box plots showing 20α-OHP levels significantly increased in the TKO mice between 37-80 %lifetime and declined in advanced stages (81-100 %lifetime). The mean, median (indicated by a small square), potential outliers, minimum, maximum, and 1.5 interquartile range are shown. Raw data were autoscaled. Fold changes were calculated as the base 2 logarithm of the 20α-OHP ratios of TKO / TKO ctrl samples. Positive values indicate higher serum levels in TKO animals, and negative values indicate lower serum levels in TKO animals compared to TKO controls.

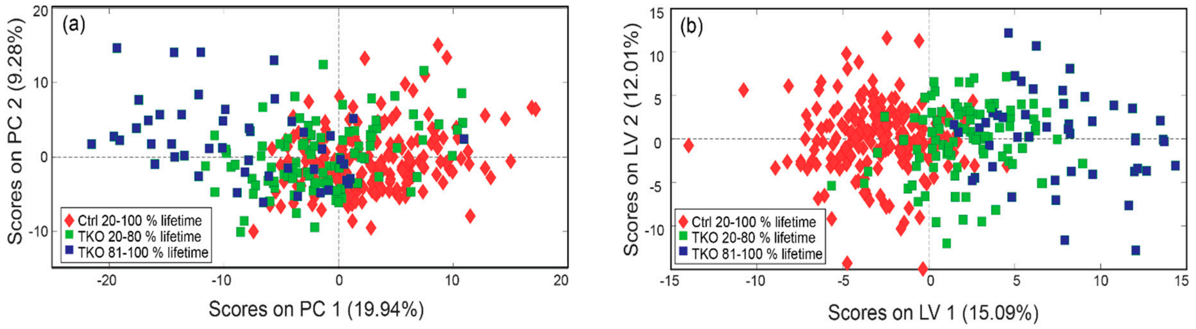

Figure S9. Unsupervised and supervised multivariate analysis of statistically-significant (-) HILIC UHPLC-MS compounds. (a) PCA score plot showing clustering of TKO and TKO control samples using statistically significant features selected with Welch's t-test with a Benjamini-Hochberg correction. (b) oPLS-DA score plot showing clustering of TKO and TKO control samples based on the abundance of statistically significant-features. TKO samples at 20-80 %lifetime are represented with green squares. TKO samples at 81-100 %lifetime are shown as blue squares, and TKO control samples at 20-100 %lifetime are shown as red diamonds.

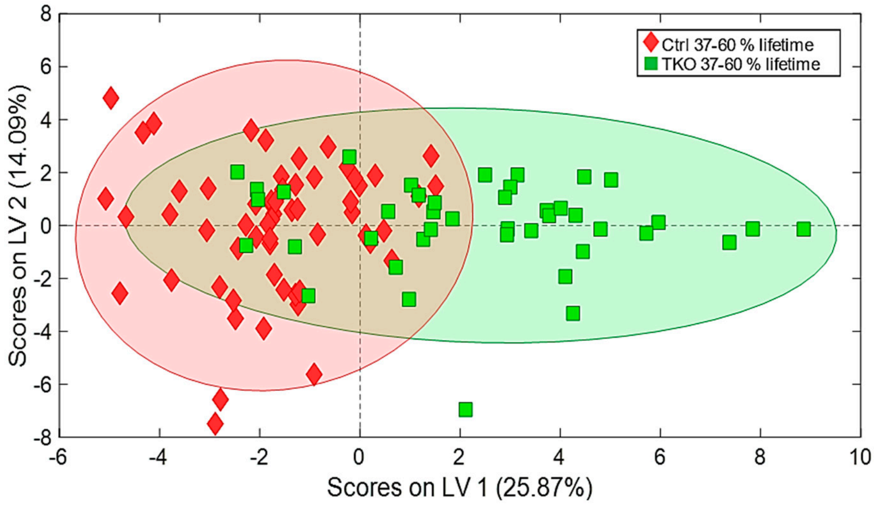

Figure S10. Supervised multivariate analysis of (-) HILIC early stage data. oPLS-DA score plot showing clustering of early-stage TKO and TKO controls at 37-60 %lifetime using only the abundances of 46 statistically significant features ($p<0.05$) detected with (-) HILIC UHPLC-MS. TKO samples are shown as green squares and TKO control samples are red diamonds. Cross-validated sensitivity and specificity were 84.5% and 69.0%, respectively.

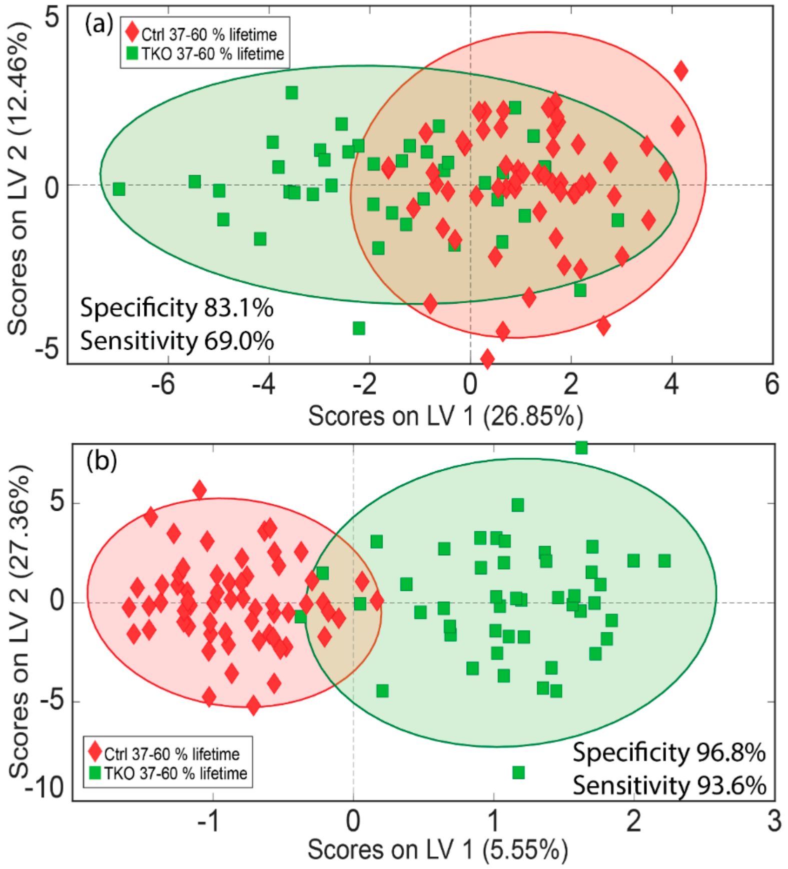

Figure S11. Supervised oPLS-DA multivariate analysis of optimized feature sets. Score plots show differences between TKO and TKO controls for serum samples collected at 37-60 %lifetime. Plot (a) shows supervised analysis of the RP UHPLC-MS 22-feature panel, (b) shows clusters of statistically-significant ($q<0.05$) amino acid, TCA acid, bile acid, and arachidonic acid metabolite data. For all features, q-values were calculated with a two-tailed Welch's t-test with Benjamini-Hochberg correction.

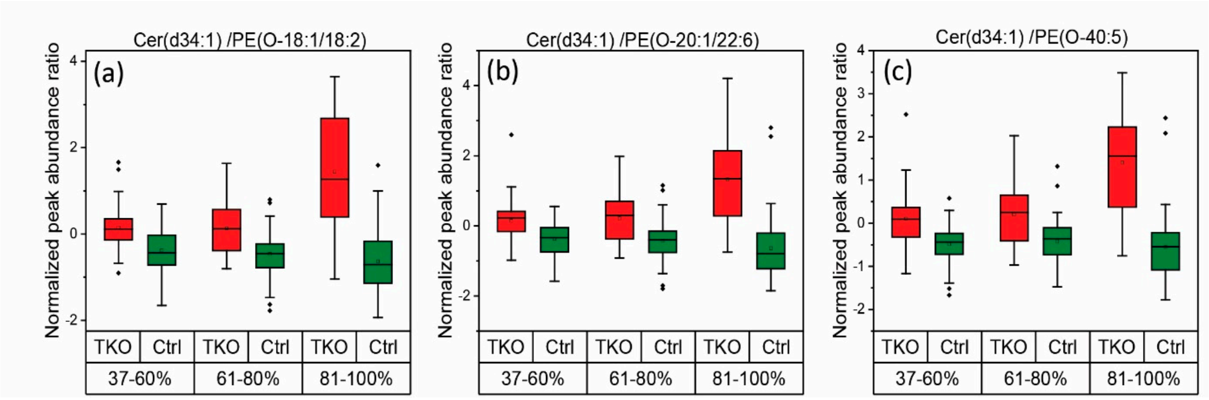

Figure S12. Comparison of serum lipid ratios for various %lifetimes in TKO and TKO control animals. The mean, median (indicated by a small square), potential outliers, minimum, maximum, and 1.5 interquartile range are shown. The data were G-log transformed and autoscaled in MetaboAnalyst prior to these calculations.

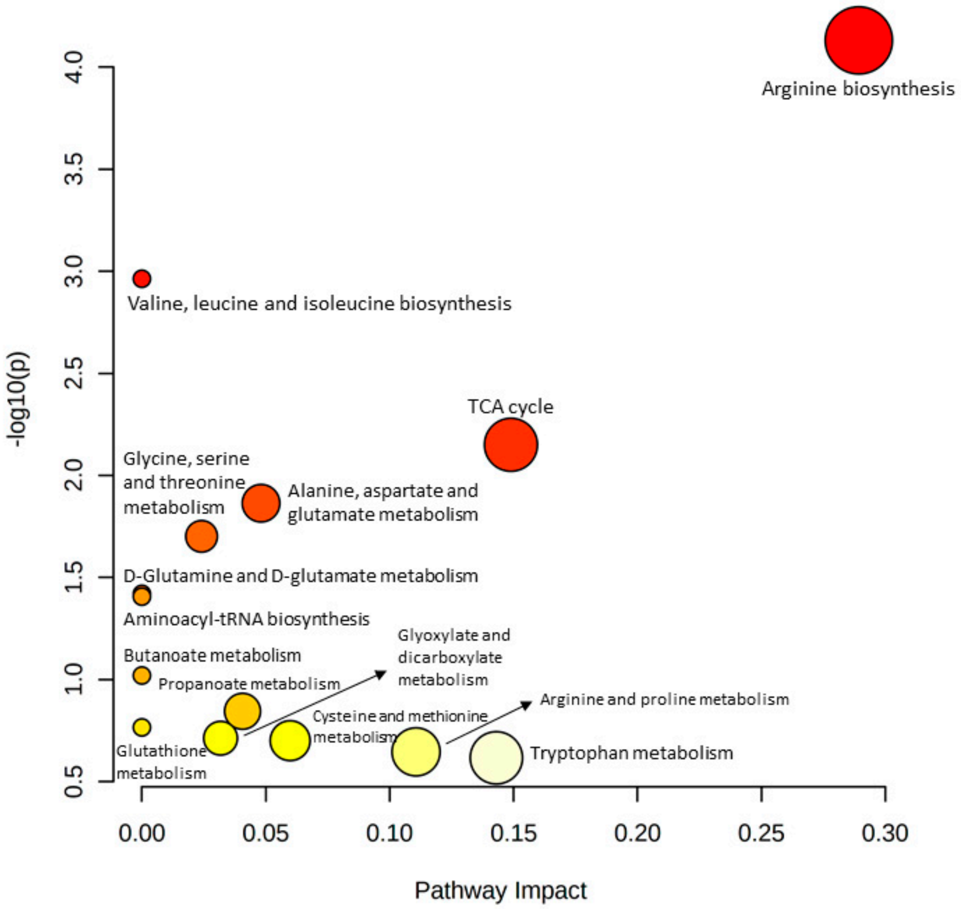

Figure S13. Pathway analysis of HILIC data. Pathway enrichment results were generated using the Metabolomic Pathway Analysis (MetPA) tool in the MetaboAnalyst web portal. Features statistically significant (q<0.05) between TKO and TKO controls at 36-60 %lifetime were used for analysis. The color scale, with red being the highest and white being the lowest, represents the pathway impact values. The size of the bubbles represents the p-values obtained from pathway enrichment analysis.

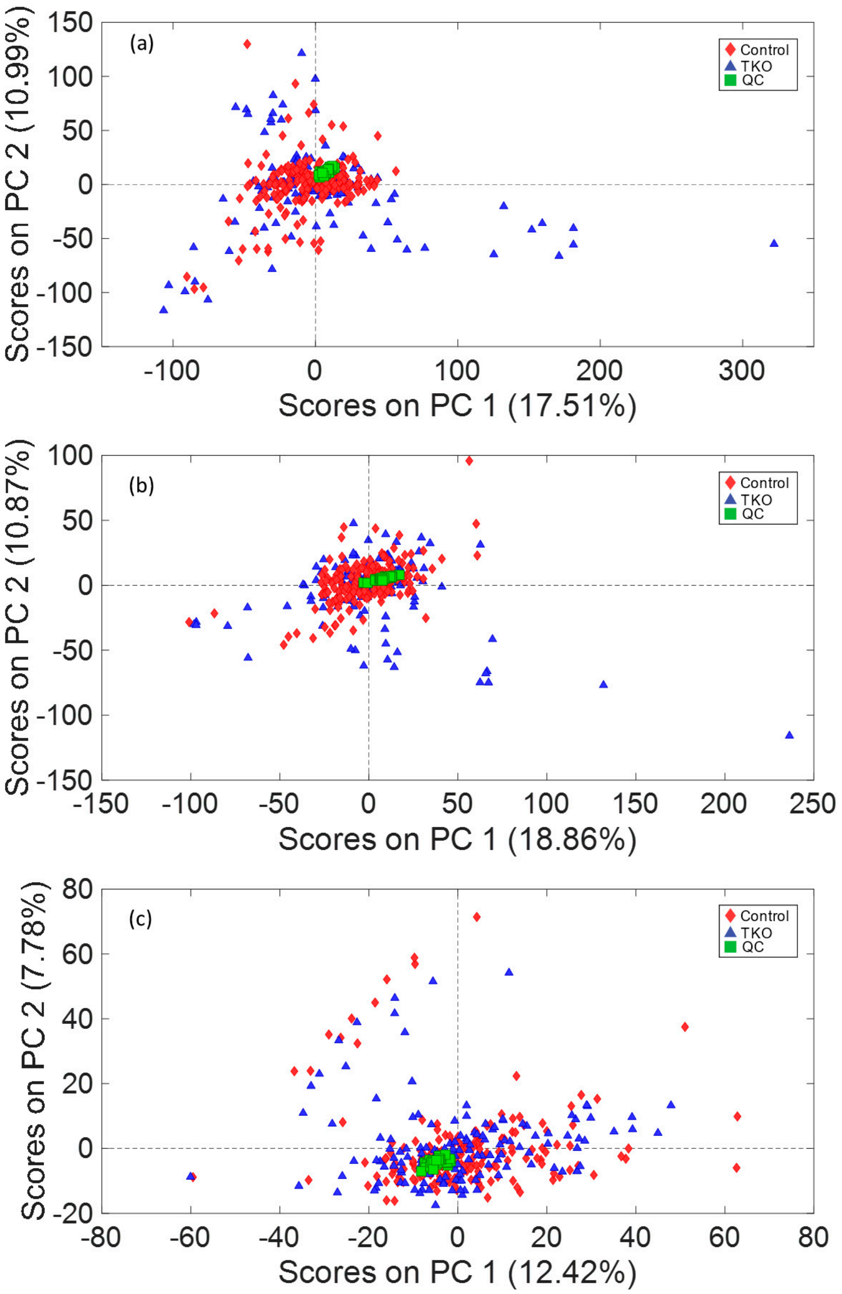

Figure S14. Unsupervised Principal Component Analysis (PCA). Score plot of TKO, TKO control, and pooled quality control (QC) samples using (a) all 11,384 compounds detected in (+) positive ion mode reverse phase (RP) UHPLC-MS, (b) 3,475 compounds detected in (-) negative ion mode RP UHPLC-MS, and (c) 1,988 compounds detected in (-) negative ion mode HILIC UHPLC-MS experiments. TKO samples, TKO controls, and QC are shown with blue triangles, red diamonds, and green squares, respectively. Tight clustering of QC injections was observed in the (+) RP and (-) RP, and (-) HILIC datasets, indicating minimal instrument drift and excellent data reproducibility through the course of all experiments.

Table S1. Composition of internal standards used in UHPLC-MS. (a) Stable isotope-labeled chemical standards used for preparing the internal standard (IS) mixture for RP UHPLC-MS lipidomics; (b) stable isotope-labeled chemical standards used for preparing the polar metabolite IS mixture for HILIC UHPLC-MS. Isotopically labeled lipid standards for the lipidomic IS mixture were purchased from Avanti Polar Lipids (Alabaster, AL). Isotopically labeled polar metabolite standards were purchased from Cambridge Isotope Laboratories (Tewksbury, MA) and were used to prepare the IS mixture for HILIC experiments.

(a) (b)

**Isotopically labeled lipids**	**CAS number**	**Concentration in stock solution**	**Isotopically labeled polar metabolites**	**CAS number**	**Concentration in stock solution**
LPC (18:1(d7))	2097561-13-0	47.3 μM	$^{13}C_6$ arginine	201740-91-2	808.0 μM
LPE(18:1(d7))	2260669-47-2	10.3 μM	$^{13}C_5$ hypoxanthine	N/A	39.7 μM
PC (15:0/18:1(d7))	2097561-16-3	212.4 μM	hippuric acid D_5	53518-98-2	256.0 μM
PE (15:0/18:1(d7))	2097561-15-2	7.03 μM	^{13}C methionine D_3	263568-73-6	212.0 μM
PS (15:0/18:1(d7))	2260669-40-5	6.43 μM			
PG (15:0/18:1(d7))	2260669-42-7	39.3 μM			
PI (15:0/18:1(d7))	2260669-44-9	11.8 μM			
CE (18:1(d7))	1416275-35-8	531.8 μM			
DG (15:0/18:1(d7))	2097561-14-1	588.0 μM			
TG (15:0/18:1(d7)/15:0)	2097561-17-4	812.3 μM			
SM (18:1(d9))	2260669-50-7	40.6 μM			
cholesterol-d7	83199-47-7	254.0 μM			

Table S2. Chromatographic gradients for RP and HILIC methods. For RP separations, mobile phase A was 10 mM ammonium acetate with water/acetonitrile (40:60 v/v) and mobile phase B was 10 mM ammonium acetate with 2-isopropanol/acetonitrile (90:10 v/v). 0.1% formic acid was added to both mobile phases in the RP experiments. Mobile phase A for HILIC was water/acetonitrile (80:20 v/v), 10 mM ammonium formate and 0.1% formic acid and mobile phase B was acetonitrile with 0.1% formic acid. The column temperature was held at 50 ⁰C for the RP and held at 40 ⁰C for the HILIC.

**RP UHPLC Gradient**				**HILIC UHPLC Gradient**			
**Time (min)**	**Mobile phase A**	**Mobile phase B**	**Flow rate (ml min^{-1})**	**Time (min)**	**Mobile phase A**	**Mobile phase B**	**Flow rate (ml min^{-1})**
0.0	80%	20%	0.4	0.0	5%	95%	0.4
0.0	80%	20%	0.4	0.5	5%	95%	0.4
1.0	40%	60%	0.4	8.0	60%	40%	0.4
5.0	30%	70%	0.4	9.4	60%	40%	0.4
5.5	15%	85%	0.4	9.5	5%	95%	0.4
8.0	10%	90%	0.4	12.0	5%	95%	0.4
8.2	0%	100%	0.4				
10.5	0%	100%	0.4				
10.7	80%	20%	0.4				
12.0	80%	20%	0.4				

Table S3. MS parameters. (a) RP UHPLC-MS and (b) HILIC UHPLC-MS.

**(a) MS parameters for RP UHPLC**			**(b) MS parameters for HILIC UHPLC**		
**MS parameter**	**Positive mode**	**Negative mode**	**MS parameter**	**Positive mode**	**Negative mode**
Capillary temperature	275 °C	275 °C	Capillary temperature	275 °C	275 °C
Spray voltage	+3.0 kV	-2.8 kV	Spray voltage	+3.5 kV	-2.5kV
Sheath gas flow rate	60 Arb.	60 Arb.	Sheath gas flow rate	40 Arb.	40 Arb.
Auxiliary gas flow rate	18 Arb.	18 Arb.	Auxiliary gas flow rate	8 Arb.	8 Arb.
Sweep gas flow rates	4 Arb.	4 Arb.	Sweep gas flow rates	1 Arb.	1 Arb.
Probe Heater Temperature	425 °C	425 °C	Probe Heater Temperature	320 °C	320 °C

Arb: Arbitrary units.

Table S4. Serial serum collection information. The mouse ID, date of serum collection, mouse age in weeks and the corresponding %lifetime values are given for each mouse used in the analysis.

Phenotype	Mouse ID	LC-MS Sample ID	Mouse Age (weeks)	%Lifetime	Collection Date (MM/DD/YY)
TKO	7320	T1_1	8	25.0%	5/10/2019
TKO	7320	T1_2	10	31.0%	5/24/2019
TKO	7320	T1_3	12	38.0%	6/7/2019
TKO	7320	T1_4	14	44.0%	6/21/2019
TKO	7320	T1_5	16	50.0%	7/5/2019
TKO	7320	T1_6	18	56.0%	7/19/2019
TKO	7320	T1_7	20	63.0%	8/2/2019
TKO	7320	T1_8	22	69.0%	8/16/2019
TKO	7320	T1_9	24	75.0%	8/30/2019
TKO	7320	T1_10	26	81.0%	9/13/2019
TKO	7320	T1_11	28	88.0%	9/27/2019
TKO	7320	T1_12	30	94.0%	10/11/2019
TKO	7320	T1_13	32	100.0%	10/25/2019
TKO	7390	T4_1	8	33.0%	5/31/2019
TKO	7390	T4_2	10	42.0%	6/14/2019
TKO	7390	T4_3	12	50.0%	6/28/2019
TKO	7390	T4_4	14	58.0%	7/12/2019
TKO	7390	T4_5	16	67.0%	7/26/2019
TKO	7390	T4_6	18	75.0%	8/9/2019
TKO	7390	T4_7	20	83.0%	8/23/2019
TKO	7390	T4_8	22	92.0%	9/6/2019
TKO	7390	T4_9	24	100.0%	9/20/2019
TKO	7453	T5_1	8	33.0%	5/31/2019
TKO	7453	T5_2	10	42.0%	6/14/2019
TKO	7453	T5_3	12	50.0%	6/28/2019
TKO	7453	T5_4	14	58.0%	7/12/2019
TKO	7453	T5_5	16	67.0%	7/26/2019
TKO	7453	T5_6	18	75.0%	8/9/2019
TKO	7453	T5_7	20	83.0%	8/23/2019
TKO	7453	T5_8	22	92.0%	9/6/2019
TKO	7453	T5_9	24	100.0%	9/20/2019
TKO	8097	T7_1	8	33.0%	8/30/2019
TKO	8097	T7_2	10	42.0%	9/13/2019
TKO	8097	T7_3	12	50.0%	9/27/2019
TKO	8097	T7_4	14	58.0%	10/11/2019
TKO	8097	T7_5	16	67.0%	10/25/2019
TKO	8097	T7_6	18	75.0%	11/8/2019
TKO	8097	T7_7	20	83.0%	11/22/2019
TKO	8097	T7_8	22	92.0%	12/6/2019
TKO	8097	T7_9	24	100.0%	12/20/2019
TKO	8098	T8_1	8	36.0%	8/30/2019
TKO	8098	T8_2	10	45.0%	9/13/2019
TKO	8098	T8_3	12	55.0%	9/27/2019
TKO	8098	T8_4	14	64.0%	10/11/2019

TKO	8098	T8_5	16	73.0%	10/25/2019
TKO	8098	T8_6	18	82.0%	11/8/2019
TKO	8098	T8_7	20	91.0%	11/22/2019
TKO	8098	T8_8	22	100.0%	12/6/2019
TKO	8100	T9_1	8	33.0%	8/30/2019
TKO	8100	T9_2	10	42.0%	9/13/2019
TKO	8100	T9_3	12	50.0%	9/27/2019
TKO	8100	T9_4	14	58.0%	10/11/2019
TKO	8100	T9_5	16	67.0%	10/25/2019
TKO	8100	T9_6	18	75.0%	11/8/2019
TKO	8100	T9_7	20	83.0%	11/22/2019
TKO	8100	T9_8	22	92.0%	12/6/2019
TKO	8100	T9_9	24	100.0%	12/20/2019
TKO	8104	T10_1	8	29.0%	8/30/2019
TKO	8104	T10_2	10	36.0%	9/13/2019
TKO	8104	T10_3	12	43.0%	9/27/2019
TKO	8104	T10_4	14	50.0%	10/11/2019
TKO	8104	T10_5	16	57.0%	10/25/2019
TKO	8104	T10_6	18	64.0%	11/8/2019
TKO	8104	T10_7	20	71.0%	11/22/2019
TKO	8104	T10_8	22	79.0%	12/6/2019
TKO	8104	T10_9	24	86.0%	12/20/2019
TKO	8104	T10_10	26	93.0%	1/3/2020
TKO	8104	T10_11	28	100.0%	1/17/2020
TKO	8256	T11_1	8	22.0%	9/20/2019
TKO	8256	T11_2	10	28.0%	10/4/2019
TKO	8256	T11_3	12	33.0%	10/18/2019
TKO	8256	T11_4	14	39.0%	11/1/2019
TKO	8256	T11_5	16	44.0%	11/15/2019
TKO	8256	T11_6	18	50.0%	11/29/2019
TKO	8256	T11_7	20	56.0%	12/13/2019
TKO	8256	T11_8	22	61.0%	12/27/2019
TKO	8256	T11_9	24	67.0%	1/10/2020
TKO	8256	T11_10	26	72.0%	1/24/2020
TKO	8256	T11_11	28	78.0%	2/7/2020
TKO	8256	T11_12	30	83.0%	2/20/2020
TKO	8256	T11_13	32	89.0%	3/5/2020
TKO	8256	T11_14	34	94.0%	3/19/2020
TKO	8256	T11_15	36	100.0%	4/2/2020
TKO	8288	T12_1	8	31.0%	9/27/2019
TKO	8288	T12_2	10	38.0%	10/11/2019
TKO	8288	T12_3	12	46.0%	10/25/2019
TKO	8288	T12_4	14	54.0%	11/8/2019
TKO	8288	T12_5	16	62.0%	11/22/2019
TKO	8288	T12_6	18	69.0%	12/6/2019
TKO	8288	T12_7	20	77.0%	12/20/2019
TKO	8288	T12_8	22	85.0%	1/3/2020
TKO	8288	T12_9	24	92.0%	1/17/2020

TKO	8288	T12_10	26	100.0%	1/31/2020
TKO	8465	T13_1	8	33.0%	11/1/2019
TKO	8465	T13_2	10	42.0%	11/15/2019
TKO	8465	T13_3	12	50.0%	11/29/2019
TKO	8465	T13_4	14	58.0%	12/13/2019
TKO	8465	T13_5	16	67.0%	12/27/2019
TKO	8465	T13_6	18	75.0%	1/10/2020
TKO	8465	T13_7	20	83.0%	1/24/2020
TKO	8465	T13_8	22	92.0%	2/7/2020
TKO	8465	T13_9	24	100.0%	2/20/2020
TKO	8470	T14_1	8	36.0%	11/1/2019
TKO	8470	T14_2	10	45.0%	11/15/2019
TKO	8470	T14_3	12	55.0%	11/29/2019
TKO	8470	T14_4	14	64.0%	12/13/2019
TKO	8470	T14_5	16	73.0%	12/27/2019
TKO	8470	T14_6	18	82.0%	1/10/2020
TKO	8470	T14_7	20	91.0%	1/24/2020
TKO	8470	T14_8	22	100.0%	2/7/2020
TKO	8472	T15_1	8	27.0%	11/1/2019
TKO	8472	T15_2	10	33.0%	11/15/2019
TKO	8472	T15_3	12	40.0%	11/29/2019
TKO	8472	T15_4	14	47.0%	12/13/2019
TKO	8472	T15_5	16	53.0%	12/27/2019
TKO	8472	T15_6	18	60.0%	1/10/2020
TKO	8472	T15_7	20	67.0%	1/24/2020
TKO	8472	T15_8	22	73.0%	2/7/2020
TKO	8472	T15_9	24	80.0%	2/20/2020
TKO	8472	T15_10	26	87.0%	3/5/2020
TKO	8472	T15_11	28	93.0%	3/19/2020
TKO	8472	T15_12	30	100.0%	4/3/2020
TKO	8747	T17_1	8	25.0%	1/3/2020
TKO	8747	T17_2	10	31.0%	1/17/2020
TKO	8747	T17_3	12	38.0%	1/31/2020
TKO	8747	T17_4	14	44.0%	2/14/2020
TKO	8747	T17_5	16	50.0%	2/27/2020
TKO	8747	T17_6	18	56.0%	3/12/2020
TKO	8747	T17_7	20	63.0%	3/26/2020
TKO	8747	T17_8	22	69.0%	4/10/2020
TKO	8747	T17_9	24	75.0%	4/23/2020
TKO	8747	T17_10	26	81.0%	5/7/2020
TKO	8747	T17_11	28	88.0%	5/19/2020
TKO	8747	T17_12	30	94.0%	6/4/2020
TKO	8747	T17_13	32	100.0%	6/18/2020
TKO	7245	T3_1	8	31.0%	5/10/2019
TKO	7245	T3_2	10	38.0%	5/24/2019
TKO	7245	T3_3	12	46.0%	6/7/2019
TKO	7245	T3_4	14	54.0%	6/21/2019
TKO	7245	T3_5	16	62.0%	7/5/2019

TKO	7245	T3_6	18	69.0%	7/19/2019
TKO	7245	T3_7	20	77.0%	8/2/2019
TKO	7245	T3_8	22	85.0%	8/16/2019
TKO	7245	T3_9	24	92.0%	8/30/2019
TKO	7245	T3_10	26	100.0%	9/13/2019
TKO	7529	T6_1	8	31.0%	6/14/2019
TKO	7529	T6_2	10	38.0%	6/28/2019
TKO	7529	T6_3	12	46.0%	7/12/2019
TKO	7529	T6_4	14	54.0%	7/26/2019
TKO	7529	T6_5	16	62.0%	8/9/2019
TKO	7529	T6_6	18	69.0%	8/23/2019
TKO	7529	T6_7	20	77.0%	9/6/2019
TKO	7529	T6_8	22	85.0%	9/20/2019
TKO	7529	T6_9	24	92.0%	10/4/2019
TKO	7529	T6_10	26	100.0%	10/18/2019
Control	7244	C1_1	8	20.0%	5/10/2019
Control	7244	C1_2	10	25.0%	5/24/2019
Control	7244	C1_3	12	30.0%	6/7/2019
Control	7244	C1_4	14	35.0%	6/21/2019
Control	7244	C1_5	16	40.0%	7/5/2019
Control	7244	C1_6	18	45.0%	7/19/2019
Control	7244	C1_7	20	50.0%	8/2/2019
Control	7244	C1_8	22	55.0%	8/16/2019
Control	7244	C1_9	24	60.0%	8/30/2019
Control	7244	C1_10	26	65.0%	9/13/2019
Control	7244	C1_11	28	70.0%	9/27/2019
Control	7244	C1_12	30	75.0%	10/11/2019
Control	7244	C1_13	32	80.0%	10/25/2019
Control	7244	C1_14	34	85.0%	11/8/2019
Control	7244	C1_15	36	90.0%	11/22/2019
Control	7244	C1_16	38	95.0%	12/6/2019
Control	7244	C1_17	40	100.0%	12/20/2019
Control	7527	C3_1	8	33.0%	6/14/2019
Control	7527	C3_2	10	42.0%	6/28/2019
Control	7527	C3_3	12	50.0%	7/12/2019
Control	7527	C3_4	14	58.0%	7/26/2019
Control	7527	C3_5	16	67.0%	8/9/2019
Control	7527	C3_6	18	75.0%	8/23/2019
Control	7527	C3_7	20	83.0%	9/6/2019
Control	7527	C3_8	22	92.0%	9/20/2019
Control	7527	C3_9	24	100.0%	10/4/2019
Control	7531	C4_1	8	21.0%	6/14/2019
Control	7531	C4_2	10	26.0%	6/28/2019
Control	7531	C4_3	12	32.0%	7/12/2019
Control	7531	C4_4	14	37.0%	7/26/2019
Control	7531	C4_5	16	42.0%	8/9/2019
Control	7531	C4_6	18	47.0%	8/23/2019
Control	7531	C4_7	20	53.0%	9/6/2019

Control	7531	C4_8	22	58.0%	9/20/2019
Control	7531	C4_9	24	63.0%	10/4/2019
Control	7531	C4_10	26	68.0%	10/18/2019
Control	7531	C4_11	28	74.0%	11/1/2019
Control	7531	C4_12	30	79.0%	11/15/2019
Control	7531	C4_13	32	84.0%	11/27/2019
Control	7531	C4_14	34	89.0%	12/13/2019
Control	7531	C4_15	36	95.0%	12/27/2019
Control	7531	C4_16	38	100.0%	1/17/2020
Control	7532	C5_1	8	21.0%	6/14/2019
Control	7532	C5_2	10	26.0%	6/28/2019
Control	7532	C5_3	12	32.0%	7/12/2019
Control	7532	C5_4	14	37.0%	7/26/2019
Control	7532	C5_5	16	42.0%	8/9/2019
Control	7532	C5_6	18	47.0%	8/23/2019
Control	7532	C5_7	20	53.0%	9/6/2019
Control	7532	C5_8	22	58.0%	9/20/2019
Control	7532	C5_9	24	63.0%	10/4/2019
Control	7532	C5_10	26	68.0%	10/18/2019
Control	7532	C5_11	28	74.0%	11/1/2019
Control	7532	C5_12	30	79.0%	11/15/2019
Control	7532	C5_13	32	84.0%	11/27/2019
Control	7532	C5_14	34	89.0%	12/13/2019
Control	7532	C5_15	36	95.0%	12/27/2019
Control	7532	C5_16	38	100.0%	1/17/2020
Control	7616	C6_1	8	29.0%	6/21/2019
Control	7616	C6_2	10	36.0%	7/5/2019
Control	7616	C6_3	12	43.0%	7/19/2019
Control	7616	C6_4	14	50.0%	8/2/2019
Control	7616	C6_5	16	57.0%	8/16/2019
Control	7616	C6_6	18	64.0%	8/30/2019
Control	7616	C6_7	20	71.0%	9/13/2019
Control	7616	C6_8	22	79.0%	9/27/2019
Control	7616	C6_9	24	86.0%	10/11/2019
Control	7616	C6_10	26	93.0%	10/25/2019
Control	7616	C6_11	28	100.0%	11/8/2019
Control	7781	C7_1	8	24.0%	7/12/2019
Control	7781	C7_2	10	29.0%	7/26/2019
Control	7781	C7_3	12	35.0%	8/9/2019
Control	7781	C7_4	14	41.0%	8/23/2019
Control	7781	C7_5	16	47.0%	9/6/2019
Control	7781	C7_6	18	53.0%	9/20/2019
Control	7781	C7_7	20	59.0%	10/4/2019
Control	7781	C7_8	22	65.0%	10/18/2019
Control	7781	C7_9	24	71.0%	11/1/2019
Control	7781	C7_10	26	76.0%	11/15/2019
Control	7781	C7_11	28	82.0%	11/29/2019
Control	7781	C7_12	30	88.0%	12/13/2019

Control	7781	C7_13	32	94.0%	12/27/2019
Control	7781	C7_14	34	100.0%	1/17/2020
Control	8469	C12_1	8	27.0%	11/1/2019
Control	8469	C12_2	10	33.0%	11/15/2019
Control	8469	C12_3	12	40.0%	11/29/2019
Control	8469	C12_4	14	47.0%	12/13/2019
Control	8469	C12_5	16	53.0%	12/27/2019
Control	8469	C12_6	18	60.0%	1/10/2020
Control	8469	C12_7	20	67.0%	1/24/2020
Control	8469	C12_8	22	73.0%	2/7/2020
Control	8469	C12_9	24	80.0%	2/20/2020
Control	8469	C12_10	26	87.0%	3/5/2020
Control	8469	C12_11	28	93.0%	3/19/2020
Control	8469	C12_12	30	100.0%	4/3/2020
Control	8103	C8_1	8	24.0%	8/30/2019
Control	8103	C8_2	10	29.0%	9/13/2019
Control	8103	C8_3	12	35.0%	9/27/2019
Control	8103	C8_4	14	41.0%	10/11/2019
Control	8103	C8_5	16	47.0%	10/25/2019
Control	8103	C8_6	18	53.0%	11/8/2019
Control	8103	C8_7	20	59.0%	11/22/2019
Control	8103	C8_8	22	65.0%	12/6/2019
Control	8103	C8_9	24	71.0%	12/20/2019
Control	8103	C8_10	26	76.0%	1/3/2020
Control	8103	C8_11	28	82.0%	1/17/2020
Control	8103	C8_12	30	88.0%	1/31/2020
Control	8103	C8_13	32	94.0%	2/14/2020
Control	8103	C8_14	34	100.0%	2/21/2020
Control	8257	C9_1	8	21.0%	9/20/2019
Control	8257	C9_2	10	26.0%	10/4/2019
Control	8257	C9_3	12	32.0%	10/18/2019
Control	8257	C9_4	14	37.0%	11/1/2019
Control	8257	C9_5	16	42.0%	11/15/2019
Control	8257	C9_6	18	47.0%	11/29/2019
Control	8257	C9_7	20	53.0%	12/13/2019
Control	8257	C9_8	22	58.0%	12/27/2019
Control	8257	C9_9	24	63.0%	1/10/2020
Control	8257	C9_10	26	68.0%	1/24/2020
Control	8257	C9_11	28	74.0%	2/7/2020
Control	8257	C9_12	30	79.0%	2/20/2020
Control	8257	C9_13	32	84.0%	3/5/2020
Control	8257	C9_14	34	89.0%	3/19/2020
Control	8257	C9_15	36	95.0%	4/2/2020
Control	8257	C9_16	38	100.0%	4/10/2020
Control	8284	C10_1	8	27.0%	9/27/2019
Control	8284	C10_2	10	33.0%	10/11/2019
Control	8284	C10_3	12	40.0%	10/25/2019
Control	8284	C10_4	14	47.0%	11/8/2019

Control	8284	C10_5	16	53.0%	11/22/2019
Control	8284	C10_6	18	60.0%	12/6/2019
Control	8284	C10_7	20	67.0%	12/20/2019
Control	8284	C10_8	22	73.0%	1/3/2020
Control	8284	C10_9	24	80.0%	1/17/2020
Control	8284	C10_10	26	87.0%	1/31/2020
Control	8284	C10_11	28	93.0%	2/14/2020
Control	8284	C10_12	30	100.0%	2/21/2020
Control	8411	C11_1	8	25.0%	10/25/2019
Control	8411	C11_2	10	31.0%	11/8/2019
Control	8411	C11_3	12	38.0%	11/22/2019
Control	8411	C11_4	14	44.0%	12/6/2019
Control	8411	C11_5	16	50.0%	12/20/2019
Control	8411	C11_6	18	56.0%	1/3/2020
Control	8411	C11_7	20	63.0%	1/17/2020
Control	8411	C11_8	22	69.0%	1/31/2020
Control	8411	C11_9	24	75.0%	2/14/2020
Control	8411	C11_10	26	81.0%	2/27/2020
Control	8411	C11_11	28	88.0%	3/12/2020
Control	8411	C11_12	30	94.0%	3/26/2020
Control	8411	C11_13	32	100.0%	4/9/2020
Control	8512	C13_1	8	25.0%	11/15/2019
Control	8512	C13_2	10	31.0%	11/29/2019
Control	8512	C13_3	12	38.0%	12/13/2019
Control	8512	C13_4	14	44.0%	12/27/2019
Control	8512	C13_5	16	50.0%	1/10/2020
Control	8512	C13_6	18	56.0%	1/24/2020
Control	8512	C13_7	20	63.0%	2/7/2020
Control	8512	C13_8	22	69.0%	2/20/2020
Control	8512	C13_9	24	75.0%	3/5/2020
Control	8512	C13_10	26	81.0%	3/19/2020
Control	8512	C13_11	28	88.0%	4/3/2020
Control	8512	C13_12	30	94.0%	4/17/2020
Control	8512	C13_13	32	100.0%	5/1/2020
Control	8517	C14_1	8	25.0%	11/15/2019
Control	8517	C14_2	10	31.0%	11/29/2019
Control	8517	C14_3	12	38.0%	12/13/2019
Control	8517	C14_4	14	44.0%	12/27/2019
Control	8517	C14_5	16	50.0%	1/10/2020
Control	8517	C14_6	18	56.0%	1/24/2020
Control	8517	C14_7	20	63.0%	2/7/2020
Control	8517	C14_8	22	69.0%	2/20/2020
Control	8517	C14_9	24	75.0%	3/5/2020
Control	8517	C14_10	26	81.0%	3/19/2020
Control	8517	C14_11	28	88.0%	4/3/2020
Control	8517	C14_12	30	94.0%	4/17/2020
Control	8517	C14_13	32	100.0%	5/1/2020
Control	8748	C15_1	8	27.0%	1/3/2020

Control	8748	C15_2	10	33.0%	1/17/2020
Control	8748	C15_3	12	40.0%	1/31/2020
Control	8748	C15_4	14	47.0%	2/14/2020
Control	8748	C15_5	16	53.0%	2/27/2020
Control	8748	C15_6	18	60.0%	3/12/2020
Control	8748	C15_7	20	67.0%	3/26/2020
Control	8748	C15_8	22	73.0%	4/10/2020
Control	8748	C15_9	24	80.0%	4/23/2020
Control	8748	C15_10	26	87.0%	5/7/2020
Control	8748	C15_11	28	93.0%	5/19/2020
Control	8748	C15_12	30	100.0%	6/4/2020
Control	8749	C16_1	8	27.0%	1/3/2020
Control	8749	C16_2	10	33.0%	1/17/2020
Control	8749	C16_3	12	40.0%	1/31/2020
Control	8749	C16_4	14	47.0%	2/14/2020
Control	8749	C16_5	16	53.0%	2/27/2020
Control	8749	C16_6	18	60.0%	3/12/2020
Control	8749	C16_7	20	67.0%	3/26/2020
Control	8749	C16_8	22	73.0%	4/10/2020
Control	8749	C16_9	24	80.0%	4/23/2020
Control	8749	C16_10	26	87.0%	5/7/2020
Control	8749	C16_11	28	93.0%	5/19/2020
Control	8749	C16_12	30	100.0%	6/4/2020

Table S5. **Age of TKO and TKO control mice with their corresponding %lifetime values.**

%Lifetime	TKO age range (weeks), median age (weeks)	TKO Control age range (weeks), median age (weeks)
20-36%	8-12w, 8w	8-14w, 10w
37-60%	10-20w, 14w	10-24w, 16w
61-80%	14-28w, 18w	16-32w, 24w
81-100%	18-36w, 24w	20-40w, 30w

Table S6. Annotated compounds with statistically significant differential abundances in the RP dataset. The q-values were calculated using the two-tailed Welch's t-test with Benjamini-Hochberg correction for TKO and TKO controls at 20-100 %lifetime. Fold changes were calculated as the base 2 logarithm of the average abundance ratios between TKO and TKO controls. Positive values indicate higher levels in TKO animals and negative values indicate lower levels in TKO samples compared to TKO controls. The results include isomers with different fatty acid chain compositions and double bond positions. In some cases, MS/MS spectra showed fragments from two or more isomers. These features were assigned two or more annotations.

ID	Annotation	Retention Time (minutes)	Log2 fold change	q-value
3968	20 alpha-dihydroprogesterone	1.353	0.66	5.17E-07
3484	arginine	0.793	-0.25	6.88E-04
4244	Car(16:3)	1.391	-0.49	5.94E-04
4431	Car(17:1-COOH)	1.072	-0.36	3.43E-05
4359	Car(18:0)	2.322	0.3	8.08E-03
4333	Car(18:3)	1.39	-0.47	7.09E-04
4335	Car(18:3)	1.204	-0.54	1.43E-03

4418	Car(20:2)	2.146	0.42	1.25E-03
4410	Car(20:3)	1.936	0.16	1.37E-02
4481	Car(22:3)	1.554	-0.64	9.17E-08
3690	Car(4:0)	0.84	0.23	8.65E-04
4449	Car(4:0)	0.843	0.26	7.09E-04
3732	Car(5:0)	0.847	0.21	1.29E-02
3919	Car(8:0-OH)	0.871	0.22	1.92E-03
1994	CE(17:1)	6.879	0.27	1.38E-03
1624	Cer(d18:0_16:0)	5.49	0.38	1.12E-02
1784	Cer(d34:1)	5.189	0.72	3.70E-07
1618	Cer(d34:1)	5.185	0.68	9.52E-07
1931	Cer(d40:1)	7.218	0.31	7.20E-06
2108	Cer(d40:1)	7.218	0.32	1.33E-05
5144	Cer(d18:1_22:0)	6.994	0.29	2.84E-05
2001	Cer(d41:0)	7.466	0.39	5.31E-03
1987	Cer(d41:1)	7.364	0.25	3.11E-04
2282	Cer(d41:1)	7.363	0.34	2.81E-04
2209	Cer(d41:1)	7.365	0.34	8.01E-05
2158	Cer(d41:1)	7.366	0.3	1.52E-04
5208	Cer(d18:1_23:0)	7.139	0.28	2.21E-04
2044	Cer(d42:0)	7.622	0.37	2.83E-03
2111	Cer(d42:0-OH)	7.354	0.34	9.75E-03
2036	Cer(d42:1)	7.515	0.35	3.70E-07
2210	Cer(d42:1)	7.517	0.36	3.70E-07
5259	Cer(d18:1_24:0)	7.287	0.35	6.16E-07
2031	Cer(d42:2)	7.177	0.45	6.72E-07
2199	Cer(d42:2)	7.18	0.45	3.73E-06
5255	Cer(d18:1_24:1)	6.957	0.38	5.17E-07
2254	Cer(d42:2)	7.179	0.49	3.90E-06
2025	Cer(d42:3)	6.907	0.77	3.70E-07
5244	Cer(d42:3)	6.7	0.66	3.30E-06
4145	cholesterol	4.582	0.1	4.11E-03
4147	cholesterol derivative	7.885	-0.2	1.20E-04
4153	cholesterol derivative	9.572	0.37	2.35E-03
96	citrulline	1.019	-0.25	6.72E-07
4223	desmosterol	4.221	0.38	1.89E-05
4999	DG(16:0_18:1_0:0)	6.557	0.4	1.31E-03
5104	DG(16:0_18:1_0:0)	6.556	0.43	1.87E-03
4992	DG(16:0_18:2_0:0)	5.929	0.51	3.60E-04
5098	DG(16:0_18:2_0:0)	5.933	0.67	7.64E-04
5215	DG(18:0_18:2_0:0)	6.676	0.35	4.09E-03
5140	DG(18:1_18:1_0:0)	6.578	0.3	2.04E-02
5214	DG(18:1_18:1_0:0)	6.578	0.34	1.77E-02
5207	DG(18:1_18:2_0:0)	5.976	0.64	1.66E-03

5197	DG(18:2_18:2_0:0)	5.272	0.79	1.44E-03
5198	DG(18:2_18:2_0:0)	5.709	0.52	2.81E-04
5115	DG(18:2_18:3_0:0)	4.711	0.52	4.15E-03
5192	DG(18:2_18:3_0:0)	4.714	0.68	7.05E-03
5250	DG(18:2_20:1_0:0)	6.661	0.77	7.72E-05
5232	DG(18:1_20:4_0:0)	5.748	0.57	2.88E-05
5303	DG(18:1_20:4_0:0)	5.754	0.65	1.04E-04
5224	DG(18:2_20:4_0:0)	5.063	0.79	3.70E-07
5291	DG(18:2_20:4_0:0)	5.064	0.8	7.33E-07
5419	DG(18:1_22:6_0:0)	5.488	0.69	2.57E-05
185	FA(12:2)	1.215	-0.4	5.79E-03
353	FA(14:0)	2.179	-0.23	9.75E-03
580	FA(16:0-OH)	1.406	-0.24	2.26E-03
581	FA(16:0-OH)	1.556	-0.31	7.64E-04
473	FA(16:1)	2.285	-0.22	1.38E-02
736	FA(18:0-OH)	2.287	-0.28	6.66E-05
924	FA(20:1-2OH)	1.593	-0.15	2.08E-02
770	FA(20:2)	2.711	0.23	1.20E-03
837	FA(20:2-OH)	2.057	0.25	1.87E-03
838	FA(20:2-OH)	1.78	0.31	2.71E-03
828	FA(20:3-OH)	1.665	0.24	1.81E-02
816	FA(20:4-OH)	1.55	0.2	1.27E-02
3916	FA(20:5)	1.819	0.19	1.33E-02
750	FA(20:5)	1.524	0.21	1.35E-02
898	FA(22:2)	3.057	0.38	1.98E-06
889	FA(22:3)	2.817	0.38	1.20E-05
881	FA(22:4)	2.611	0.27	4.42E-05
876	FA(22:5)	2.421	0.16	8.37E-03
1032	FA(24:1)	4.009	0.28	3.35E-05
1023	FA(24:2)	3.512	0.46	1.85E-07
1146	FA(26:1)	4.774	0.19	5.89E-03
1137	FA(26:2)	2.001	-0.55	2.37E-05
1140	FA(26:2)	4.118	0.74	1.90E-06
1253	FA(28:0)	6.858	-0.21	8.35E-03
395	geranyl acetoacetate	1.354	-0.22	2.92E-04
5492	HexCer(d18:1_16:0)	4.344	0.53	6.48E-05
9617	HexCer(d34:1)	4.356	0.56	5.03E-05
2970	HexCer(d42:1)	7.227	0.25	2.03E-03
6602	HexCer(d18:1_24:0)	7	0.22	5.81E-03
2644	HexCer(d42:2) or GlcCer(d42:2)	6.888	0.25	3.40E-03
6570	HexCer(d18:1_24:1)	6.675	0.29	3.05E-03
4461	LPC(14:0)	1.515	-0.11	6.99E-03
4468	LPC(0:0_16:1)	1.729	-0.12	1.62E-02
4529	LPC(16:1_0:0)	1.74	-0.11	1.85E-02

4589	LPC(17:0)	2.196	-0.18	6.95E-03
1535	LPC(17:1)	2.342	-0.15	1.49E-03
4581	LPC(17:1)	1.948	-0.19	2.60E-03
4636	LPC(18:1)	2.143	-0.17	7.93E-04
1721	LPC(18:1)	2.342	-0.15	3.91E-04
4626	LPC(18:2_0:0)	2.117	-0.12	1.66E-02
1760	LPC(18:2)	2.04	-0.13	2.61E-03
4627	LPC(0:0_18:2)	1.851	-0.14	2.10E-04
4628	LPC(18:2)	1.769	-0.34	2.84E-07
1709	LPC(18:2)	2.04	-0.14	1.44E-03
4621	LPC(18:3)	1.608	-0.16	6.32E-03
4755	LPC(20:3_)	1.994	-0.19	1.63E-03
1814	LPC(0:0_20:3)	2.127	-0.24	5.21E-04
1815	LPC(20:3_0:0)	2.185	-0.19	8.52E-04
4736	LPC(0:0_20:4)	2.158	-0.16	1.65E-04
4737	LPC(20:4_0:0)	2.149	-0.16	2.10E-03
4738	LPC(0:0_20:4)	1.818	-0.2	1.69E-05
4739	LPC(20:4_0:0)	1.763	-0.31	6.10E-07
1808	LPC(20:4)	2.006	-0.17	4.55E-04
4719	LPC(20:5)	1.567	-0.27	7.50E-05
4866	LPC(22:6)	1.997	-0.24	2.53E-04
5068	LPC(24:2)	2.774	0.2	1.05E-02
4423	LPE(16:0)	4.564	-0.31	1.14E-02
4485	LPE(18:1)	2.183	-0.14	4.91E-03
1510	LPE(20:4)	1.992	-0.17	1.77E-02
1257	LPE(O-15:0)	2.282	-0.09	4.20E-03
4852	PC(21:0)	2.909	-0.15	2.30E-03
5521	PC(30:0)	4.116	0.24	1.35E-04
5619	PC(31:0)	5.98	0.29	1.83E-02
5711	PC(32:0)	4.852	0.16	3.05E-03
5712	PC(16:0_16:0)	3.78	0.35	3.91E-03
5678	PC(32:2)	3.699	0.55	2.30E-03
5664	PC(32:3)	3.372	0.34	1.56E-02
5808	PC(33:0)	5.105	-0.13	2.04E-02
5765	PC(33:3)	4.124	-0.18	2.33E-05
2680	PC(16:0_18:1)	5.154	0.1	1.72E-02
2606	PC(16:0_18:2)	4.562	0.11	8.35E-03
5876	PC(34:3)	3.791	0.34	3.48E-03
2593	PC(16:0_18:3)	4.146	0.16	1.78E-02
5862	PC(34:4)	3.61	0.44	6.97E-03
5863	PC(34:4)	3.459	0.32	1.46E-02
5839	PC(34:5)	3.71	0.65	2.18E-03
6052	PC(35:1)	5.23	-0.2	1.66E-03
2668	PC(17:0_18:2)	4.562	0.14	1.66E-03

6008	PC(35:3)	4.539	0.52	9.28E-04
6250	PC(36:0)	6.533	-0.2	1.44E-03
2766	PC(18:0_18:0)	6.794	-0.17	3.31E-03
10151	PC(36:4)	4.223	-0.06	1.54E-02
6457	PC(36:4)	4.217	-0.08	1.58E-02
6101	PC(36:5)	3.516	0.56	1.77E-02
6083	PC(36:6)	4.606	0.5	1.91E-03
10064	PC(37:4)	4.183	0.34	1.90E-03
6343	PC(37:4)	4.18	0.29	4.00E-03
6346	PC(37:4)	4.474	-0.15	6.80E-03
6308	PC(37:5)	5.265	0.37	6.12E-03
6309	PC(37:5)	5.6	-0.14	2.01E-02
6312	PC(37:5)	5	0.12	1.71E-02
10012	PC(37:6)	3.775	0.51	1.17E-03
11142	PC(37:6)	3.761	0.5	1.46E-03
6273	PC(37:6)	3.762	0.49	1.76E-03
6624	PC(38:2)	5.9	0.32	4.73E-03
6599	PC(38:3)	6.547	-0.32	1.01E-05
6566	PC(38:4)	4.54	0.22	2.60E-03
2946	PC(16:0_22:4)	4.714	0.3	4.16E-04
2934	PC(16:0_22:5)	4.727	0.22	1.40E-02
2850	PC(16:0_22:5)	4.731	0.2	1.60E-02
6494	PC(38:6)	4.411	-0.11	3.71E-04
6496	PC(38:6)	4.058	0.22	7.55E-04
2843	[a]PC(16:0_22:6) and PC(18:0_20:6)	4.268	0.18	3.05E-03
6460	PC(38:7)	3.548	0.39	8.87E-03
6804	PC(39:3)	5.79	-0.12	1.55E-02
6735	PC(39:5)	4.609	0.33	8.63E-03
407	PC(40:2)	1.204	0.15	1.54E-02
7038	PC(40:2)	6.545	0.15	1.81E-02
6995	PC(40:4)	5.593	0.34	9.83E-04
6996	PC(40:4)	5.357	0.27	2.73E-03
6997	PC(40:4)	5.221	0.44	2.12E-04
6960	PC(40:5)	5.903	0.3	5.99E-03
6961	PC(40:5)	5.336	0.38	1.46E-03
6962	PC(40:5)	4.694	0.52	3.02E-04
6922	PC(40:6)	4.801	0.15	2.10E-02
6923	PC(40:6)	3.771	0.39	5.34E-03
6924	PC(40:6)	4.368	0.64	2.30E-05
3055	PC(18:0_22:6)	5.012	0.17	5.86E-03
6883	PC(40:7)	4.738	0.35	1.13E-03
6885	PC(40:7)	3.816	0.46	3.50E-03
6863	PC(40:8)	3.655	0.3	1.20E-02
6825	PC(40:9)	3.35	0.43	7.24E-03

7098	PC(41:6)	5.228	0.28	4.05E-04
7075	PC(41:7)	4.437	0.28	3.99E-03
7322	PC(42:4)	6.267	-0.21	3.17E-04
7295	PC(42:5)	5.587	0.47	2.93E-04
7296	PC(42:5)	5.71	0.35	9.88E-03
7262	PC(42:6)	5.688	0.21	1.91E-02
7263	PC(42:6)	5.86	0.2	9.20E-03
7240	PC(42:7)	4.793	0.42	7.90E-05
10493	PC(42:8)	4.108	0.82	8.79E-07
7215	PC(42:8)	4.198	0.9	6.10E-07
7192	PC(42:9)	3.78	0.41	5.34E-03
7615	PC(44:6)	5.961	0.44	1.66E-03
7547	PC(44:8)	4.657	0.74	9.04E-06
5577	PC(O-32:2)	4.563	-0.21	9.84E-05
5696	PC(O-33:1)	6.571	-0.16	2.14E-03
5795	PC(O-34:1)	5.432	0.14	1.75E-02
2550	PC(O-18:1_16:0)	5.68	0.12	1.39E-02
2623	PC(O-34:1)	5.689	0.16	2.26E-03
5785	PC(O-34:2)	5.344	-0.12	1.54E-02
5770	PC(O-34:3)	4.709	-0.13	1.61E-02
2660	PC(O-17:1_18:2)	4.009	0.33	1.25E-03
6071	PC(O-36:0)	6.821	-0.25	7.07E-04
6054	PC(O-36:1)	6.383	-0.22	1.86E-04
6033	PC(O-36:2)	5.727	-0.2	2.27E-04
6034	PC(O-36:2)	5.544	0.25	2.36E-03
6011	PC(O-36:3)	5.576	-0.21	2.91E-04
6012	PC(O-36:3)	4.858	0.12	8.87E-03
5987	PC(O-36:4)	4.266	0.16	2.04E-02
6104	PC(O-37:5)	4.965	-0.27	4.57E-04
6105	PC(O-37:5)	4.955	-0.25	1.13E-03
6406	PC(O-38:2)	6.551	-0.28	6.10E-07
6377	PC(O-38:3)	5.927	-0.18	2.18E-03
67	PC(O-38:4)	1.017	0.1	1.78E-02
6349	PC(O-38:4)	5.062	0.13	6.00E-03
6314	PC(O-38:5)	5.404	-0.26	7.77E-06
6315	PC(O-38:5)	4.427	-0.19	1.35E-03
2853	PC(O-18:1_20:4)	4.946	0.12	1.41E-02
6245	PC(O-38:7)	4.367	-0.13	1.83E-03
6810	PC(O-40:3)	6.489	-0.22	6.63E-04
10303	PC(O-40:4)	6.495	-0.3	3.70E-07
6784	PC(O-40:4)	6.476	-0.29	1.10E-06
6740	PC(O-40:5) or PC(P-40:4)	6.318	-0.5	1.02E-13
6742	PC(O-40:5)	5.293	0.19	1.20E-02
6674	PC(O-40:7)	5.174	-0.27	9.52E-07

6647	PC(O-40:8)	4.422	-0.22	5.97E-04
6621	PC(O-40:9) or PC(P-40:8)	3.898	-0.19	1.73E-03
7100	PC(O-42:6)	5.653	-0.21	3.13E-03
7103	PC(O-42:6)	6.247	-0.33	5.17E-07
6782	PC(P-40:3) or PC(O-40:4)	5.963	0.18	1.05E-02
6783	PC(P-40:3) or PC(O-40:4)	5.803	-0.19	7.09E-04
2297	PE(34:0)	6.183	0.3	8.30E-03
2294	PE(16:0_18:1)	5.359	0.4	5.81E-03
5575	PE(34:2)	4.555	0.6	5.23E-04
2288	PE(16:0_18:2)	4.75	1.54	1.13E-02
2279	PE(34:3)	4.277	0.78	4.15E-03
5568	PE(34:3)	3.924	0.56	1.14E-03
2475	PE(36:1)	6.898	0.31	3.83E-03
2366	PE(18:0_18:2)	5.578	0.44	7.60E-03
2363	PE(18:0_18:2)	4.563	0.12	6.91E-03
5781	PE(18:2_18:0)	5.382	0.43	1.30E-02
5782	PE(18:2_18:0)	5.211	0.31	1.06E-02
5766	PE(36:3)	4.592	0.44	1.66E-03
2359	PE(18:1_18:2)	4.772	1.19	8.40E-03
2355	PE(36:4)	4.246	0.54	1.27E-02
2354	PE(16:0_20:4)	4.606	0.51	6.62E-04
5747	PE(16:0_20:4)	4.052	0.39	1.39E-02
5748	PE(36:4)	4.123	-0.14	1.44E-03
5749	PE(36:4)	4.429	0.32	5.97E-04
2345	PE(36:5)	4.012	0.29	1.77E-02
2346	PE(36:5)	3.846	0.49	1.65E-02
2347	PE(36:5)	4.184	0.31	1.91E-02
5730	PE(36:5)	4.55	0.54	1.29E-04
2406	PE(17:0_20:4)	5.002	0.28	1.66E-02
6006	PE(18:0_20:3)	5.358	0.24	1.01E-02
2452	PE(18:0_20:4)	5.426	0.31	2.60E-03
5982	PE(18:0_20:4)	5.244	0.26	4.90E-03
5958	PE(38:5)	4.726	0.35	7.05E-03
2439	[b]PE(16:0_22:5) and PE(18:1_20:4)	4.649	0.28	1.05E-02
5783	PE(18:1_20:4)	4.478	0.25	1.52E-02
5934	PE(38:6)	4.601	0.36	2.30E-03
5937	PE(38:6)	4.245	0.52	6.66E-05
2434	PE(16:0_22:6)	4.432	0.67	1.89E-05
2512	PE(39:4)	5.876	0.33	1.15E-02
2492	PE(39:6)	4.783	0.98	3.93E-03
2558	PE(40:5)	5.442	0.44	1.07E-03
6274	PE(40:6)	5.027	0.37	7.64E-04
2546	PE(18:0_22:6)	5.204	0.54	3.03E-04
2537	PE(40:7)	4.473	0.6	5.63E-04

6240	PE(40:7)	5.256	0.12	1.77E-02
7154	PE(44:4)	6.042	0.42	4.15E-03
2332	PE(O-18:0_18:1)	6.796	-0.13	1.25E-02
2324	PE(O-18:0_18:2)	6.2	-0.16	2.60E-03
5656	PE(O-36:3)	4.979	-0.17	1.40E-02
2318	PE(O-18:1_18:2)	6.041	-0.19	3.18E-04
5667	PE(O-18:1_18:2)	5.848	-0.21	2.28E-05
2419	PE(O-20:0_18:2)	6.921	-0.2	1.65E-04
5867	PE(O-38:4)	5.845	-0.17	2.00E-03
2408	[c]PE(O-18:0_20:4) and PE(O-18:2_20:2)	6.039	-0.15	6.95E-03
2399	PE(O-18:1_20:4)	5.865	-0.14	7.60E-03
5570	PE(O-18:1_20:4)	5.692	-0.13	1.25E-02
5844	PE(O-18:1_20:4)	5.689	-0.17	1.66E-03
6141	PE(O-40:4)	6.417	-0.29	1.95E-05
2514	PE(O-18:0_22:4)	6.622	-0.19	4.15E-03
2503	PE(O-18:0_22:5)	6.061	-0.18	8.65E-04
6107	PE(O-18:1_22:4)	6.284	-0.22	1.88E-03
6109	PE(O-18:1_22:4)	6.526	-0.29	8.30E-07
2493	PE(O-40:6) or PE(P-40:5)	6.182	-0.2	5.09E-03
6065	PE(O-18:1_22:6)	5.441	-0.18	7.64E-04
2487	[d]PE(O-18:1_22:6) and PE(O-22:7_18:0)	5.613	-0.16	2.30E-03
2631	PE(O-20:1_22:4)	7.129	-0.35	2.10E-06
2619	PE(O-20:0_22:6)	6.674	-0.2	1.44E-03
6498	PE(O-18:1_24:5)	6.476	-0.24	6.03E-05
2607	PE(O-20:1_22:6)	6.526	-0.29	4.99E-06
2513	PE(P-18:0_22:3)	6.839	-0.2	4.84E-04
2504	PE(P-20:0_20:4)	6.73	-0.27	9.79E-06
2470	PG(18:1_18:2)	3.986	0.76	5.34E-03
2923	PI(18:0_18:1)	4.974	-0.21	1.87E-03
7279	PI(36:2)	4.569	-0.12	1.07E-02
2686	PI(16:0_20:4)	3.738	0.23	1.65E-04
2886	PI(36:4)	3.751	0.22	1.65E-04
7231	PI(36:4)	3.794	0.2	1.01E-03
7597	PI(18:0_20:3)	4.763	-0.19	2.14E-03
3176	PI(18:0_20:4)	4.356	-0.06	1.70E-02
3024	PI(18:1_20:4)	3.788	0.2	7.05E-03
7790	PI(18:1_20:4)	3.842	0.2	1.75E-02
3015	PI(16:0_22:6)	3.627	0.21	1.46E-02
2656	PS(38:3)	3.827	0.28	3.05E-03
2770	PS(18:0_22:5)	3.693	0.62	4.46E-04
2474	PS(O-36:2)	2.169	-0.19	1.72E-02
2677	PS(O-20:3_20:4)	5.869	-0.15	3.86E-03
358	ribose 5-phosphate	1.01	0.21	8.52E-04
5502	SiE(20:4)	9.282	-0.37	1.04E-07

5602	SiE(20:4)	9.282	-0.36	2.73E-07
5772	SiE(22:6)	9.06	-0.23	2.93E-04
2075	SM(d31:0)	4.754	0.1	1.89E-02
2076	SM(d31:0)	6.136	0.67	5.97E-04
5625	SM(d34:0-OH)	3.788	-0.13	1.75E-02
5590	SM(d35:1)	4.472	-0.21	3.13E-04
5569	SM(d36:1)	4.871	-0.24	2.92E-04
5691	SM(d36:1)	4.876	-0.23	2.61E-04
2543	SM(d36:1)	5.101	-0.23	1.44E-03
2491	SM(d36:1)	5.102	-0.22	6.93E-04
5675	SM(d36:2)	4.232	-0.26	1.33E-05
5791	SM(d37:1)	5.329	-0.21	1.17E-03
5904	SM(d38:1)	5.78	-0.14	8.05E-03
5886	SM(d38:2)	5.044	-0.23	1.01E-05
6044	SM(d39:1)	6.269	-0.2	1.67E-04
6175	SM(d40:2)	5.968	-0.19	3.29E-04
2804	SM(d40:2)	6.205	-0.17	4.32E-03
2733	SM(d40:2)	6.2	-0.2	2.61E-04
6419	SM(d41:1)	6.796	-0.17	1.13E-03
2819	SM(d41:1)	7.046	-0.14	2.30E-03
6388	SM(d41:2)	6.417	-0.32	5.17E-07
6389	SM(d41:2)	6.165	-0.15	1.65E-03
6614	SM(d42:2)	6.695	-0.15	1.83E-03
2892	SM(d42:2)	6.929	-0.34	1.13E-04
6855	SM(d43:1)	7.075	-0.14	3.65E-03
6819	SM(d43:2)	6.749	-0.16	2.94E-03
5943	TG(10:0_16:1_18:1)	7.565	0.68	3.62E-03
6284	TG(46:2)	7.862	0.5	2.30E-03
6224	TG(10:0_18:2_18:2)	7.322	0.62	1.81E-02
6707	TG(48:2)	7.853	0.36	7.70E-03
6708	[e]TG(14:0_16:0_18:2) and TG(14:0_16:1_18:1)	8.19	0.3	7.24E-03
6676	TG(48:3)	7.86	0.29	1.29E-02
6651	TG(12:0_18:2_18:2)	7.601	0.29	8.03E-03
6653	TG(48:4)	7.604	0.46	4.33E-03
6935	TG(49:2)	8.31	-0.3	6.19E-03
7106	[f]TG(16:0_16:1_18:1) and TG(14:0_18:1_18:1)	8.575	0.22	2.91E-03
7059	TG(16:1_16:1_18:2)	7.871	0.24	5.99E-03
7270	TG(50:4)	7.902	0.29	2.48E-03
6820	[g]TG(16:1_16:1_18:2) and TG(16:1_16:1_18:2)	7.9	0.26	3.63E-03
7061	TG(50:4)	7.905	0.29	2.52E-03
7040	TG(50:5)	7.676	0.31	6.51E-03
7244	TG(50:5)	7.674	0.31	6.03E-03
7020	TG(50:6)	7.474	0.54	1.66E-03
7403	[h]TG(16:0_18:1_18:2) and TG(16:1_18:1_18:1)	8.135	0.33	6.71E-06

7387	TG(16:1_18:1_18:2)	8.246	0.2	3.83E-03
7168	TG(16:0_18:2_18:3)	8	0.18	2.03E-02
7369	TG(52:5)	7.999	0.2	1.65E-02
7167	TG(16:1_18:2_18:2)	7.914	0.34	2.81E-04
7370	TG(52:5)	7.916	0.37	1.99E-04
7144	[i]TG(16:1_18:2_18:3) and TG(16:2_18:2_18:2)	7.683	0.29	2.63E-03
7341	TG(52:6)	7.551	0.47	3.33E-03
7345	TG(52:6)	7.693	0.33	2.82E-03
7551	TG(52:6)	7.698	0.38	3.08E-03
7323	TG(52:7)	7.514	0.35	3.05E-03
7521	TG(52:7)	7.53	0.36	2.60E-03
7627	TG(53:2)	9.087	-0.22	1.63E-02
7592	[j]TG(17:0_18:1_18:2) and TG(17:1_18:1_18:1) and TG(16:1_18:1_19:1) and TG(16:0_18:1_19:2)	8.674	-0.34	5.02E-03
7492	[k]TG(17:2_18:2_18:2) and TG(17:1_18:2_18:3)	7.819	0.31	1.77E-02
7832	[l]TG(16:0_18:1_20:1) and TG(18:0_18:1_18:1)	8.563	0.07	1.55E-02
7542	[m]TG(18:0_18:1_18:2) and TG(18:1_18:1_18:1) and TG(16:0_18:2_20:1)	8.872	-0.22	1.31E-02
7797	TG(18:1_18:1_18:1)	8.255	0.1	1.20E-02
7802	TG(54:3)	8.874	-0.26	7.55E-03
7774	TG(54:4)	8.288	0.17	1.66E-03
7769	[n]TG(18:1_18:1_18:2) and TG(18:0_18:2_18:2)	7.901	0.14	1.81E-02
7719	TG(54:6)	8.127	0.34	5.84E-06
7989	TG(54:6)	8.128	0.35	3.90E-06
7691	TG(54:7)	7.723	0.3	1.20E-02
7693	TG(18:2_18:2_18:3)	7.464	0.28	1.79E-04
7924	TG(55:6)	8.13	0.27	2.21E-04
8017	TG(18:2_18:3_20:5)	7.447	0.26	4.94E-04
8174	TG(18:0_16:0_22:6)	8.692	0.33	1.18E-02
7878	[o]TG(18:1_18:1_20:4) and TG(16:0_18:1_22:5) and TG(18:1_18:2_20:3)	8.304	0.25	1.95E-02
7879	[p]TG(18:1_18:1_20:4) and TG(16:0_18:1_22:5) and TG(18:1_18:2_20:3)	8.388	0.21	1.32E-03
8173	TG(56:6)	8.304	0.27	1.46E-02
8176	TG(56:6)	8.415	0.24	1.30E-03
7847	TG(56:7)	8.12	0.21	4.55E-04
8145	TG(56:7)	7.852	0.35	6.03E-05
8149	TG(56:7)	8.12	0.25	3.04E-04
8150	TG(56:7)	7.728	0.33	6.66E-05
8148	TG(16:0_18:1_22:6)	8.29	0.18	4.04E-03
7745	TG(56:8)	8.021	0.37	5.17E-06
8109	TG(56:8)	7.999	0.38	4.23E-06
7811	TG(18:2_18:2_20:4)	7.844	0.26	2.59E-04
8110	TG(56:8)	7.849	0.33	1.02E-04
8059	TG(56:9)	7.649	0.34	2.91E-04
8147	TG(58:10)	7.931	0.51	4.93E-03

8461	TG(58:10)	7.892	0.35	1.49E-05
8462	TG(58:10)	7.728	0.4	1.89E-05
8439	TG(58:11)	7.527	0.35	5.28E-05
8440	TG(58:11)	7.723	0.4	5.24E-05
8622	TG(58:4)	8.982	0.38	6.26E-04
8598	TG(58:5)	8.975	0.27	8.35E-03
8567	TG(58:6)	8.513	0.39	5.35E-04
8571	TG(58:6)	8.695	0.45	6.71E-06
8552	TG(18:0_18:1_22:6)	8.393	0.36	9.00E-05
8518	TG(58:8)	8.269	0.18	1.27E-02
8520	TG(18:0_20:4_20:4)	8.356	-0.24	7.58E-03
8486	TG(58:9)	7.992	0.22	2.20E-03
8555	TG(18:0_20:4_22:6)	8.025	0.53	5.35E-04
8776	TG(60:10)	7.736	0.23	2.30E-03

[a-p] For features # 2843, 2439, 2408, 2487, 6708, 7106, 6820, 7403, 7144, 7592, 7492, 7832, 7542, 7769, 7878 and 7879, the MS/MS spectra showed fragments from two or more isomers. These features were therefore assigned two or more annotations.

Table S7. Metabolite annotations for the 46 selected features in the HILIC dataset. Proposed metabolite annotation, experimental monoisotopic *m/z* value, elemental formula, chromatographic retention time (min), mass error (ppm), main adduct type detected, abundance log-transformed fold changes, and metabolite annotation confidence level are shown. Fold changes were calculated as the base 2 logarithm of the average abundance ratios between TKO and TKO controls in the 37-60 %lifetime group. Positive values indicate higher levels in TKO serum samples and negative values indicate lower levels in TKO serum samples compared to TKO controls. The confidence level for metabolite annotation was assigned as (1) exact mass, isotopic pattern, retention time, and MS/MS spectrum of a chemical standard matched to the feature. (2) exact mass, isotopic pattern, retention time, and MS/MS spectrum matched to an in-house spectral database or literature spectra (3) putative ID assignment based only on elemental formula match. (4) unknown compound. Amino acids and derivatives, TCA acids, bile acids, and arachidonic acid metabolites are shown in bold. Compound classes are provided for features with low annotation confidence levels.

ID	Elemental formula	Proposed annotation	Adduct type	Experimental *m/z*	Retention time (min)	Mass error (ppm)	Log_2 fold change TKO/ Ctrl	q-value	Annotation confidence level
52	$C_5H_6N_2$	methylpyrazine	$[M-H]^-$	93.0459	9.32	1.48	-0.27	0.03	3
**78**	**$C_4H_6O_3$**	**oxobutanoic acid**	**$[M-H]^-$**	**101.0245**	**5.11**	**0.59**	**-0.36**	**0.03**	**2**
**92**	**$C_3H_7NO_3$**	**serine**	**$[M-H]^-$**	**104.0354**	**8.71**	**0.58**	**-0.23**	**0.03**	**2**
**153**	C_8H_7N	indole	$[M-H]^-$	116.0506	6.67	0.85	-0.31	0.03	3
**159**	**$C_4H_9NO_3$**	**threonine**	**$[M-H]^-$**	**118.0509**	**8.35**	**-0.25**	**-0.23**	**0.03**	**2**
218	--	--	$[M-H]^-$	129.9365	8.09	--	0.42	0.03	4
**242**	**$C_5H_{12}N_2O_2$**	**ornithine**	**$[M-H]^-$**	**131.0827**	**8.85**	**0.77**	**-0.17**	**0.03**	**2**
**251**	**$C_4H_6O_5$**	**malic acid**	**$[M-H]^-$**	**133.0143**	**7.10**	**0.70**	**-0.47**	**0.03**	**2**
318	$C_{10}H_9N$	methylquinoline	$[M-H]^-$	142.0662	6.67	-0.35	-0.33	0.04	2
**345**	**$C_5H_6O_5$**	**oxoglutaric acid**	**$[M-H]^-$**	**145.0143**	**5.13**	**0.18**	**-0.28**	**0.04**	**2**
396	$C_7H_6O_4$	dihydroxybenzoic acid	$[M-H]^-$	153.0193	1.28	0.17	-0.56	0.02	2
532	$C_5H_5NO_4S$	hydroxypyridine sulfate	$[M-H]^-$	173.9864	8.35	-1.14	-0.5	0.03	3
**539**	**$C_6H_{13}N_3O_3$**	**citrulline**	**$[M-H]^-$**	**174.0883**	**8.85**	**-0.28**	**-0.17**	**0.03**	**2**
594	$C_6H_4N_2O_3S$	--	$[M-H]^-$	182.9867	8.66	-1.63	-0.16	0.03	4
600	$C_{10}H_{16}O_3$	acyloxy ketone derivative	$[M-H]^-$	183.1027	1.44	0.16	-0.52	0.03	2
**635**	**$C_7H_{12}N_2O_4$**	**N-acetylglutamine**	**$[M-H]^-$**	**187.0724**	**4.90**	**-0.09**	**0.26**	**0.04**	**2**
**698**	**$C_6H_8O_7$**	**citric acid/isocitric acid**	**$[M-H]^-$**	**191.0197**	**6.67**	**0.02**	**-0.23**	**0.03**	**2**
**807**	**$C_{11}H_{12}N_2O_2$**	**tryptophan**	**$[M-H]^-$**	**203.0824**	**6.67**	**-0.76**	**-0.31**	**0.03**	**2**

974	$C_{14}H_{24}O_2$	Terpenoid	$[M+H_2CO_2-H]^-$	269.1757	1.27	-0.38	-0.4	0.04	2
1083	$C_{15}H_{26}O_2$	Terpenoid	$[M-H]^-$	237.1858	1.27	-0.66	-0.67	0.03	2
1197	$C_{16}H_{26}O_2$	FA(16:3)	$[M-H]^-$	249.1858	1.27	-0.72	-0.62	0.03	2
1329	$C_{15}H_{10}O_5$	hydroxydai-dzein	$[M-H]^-$	269.0456	1.05	0.24	-0.57	0.03	2
1357	$C_{10}H_{10}O_7S$	ferulic acid sulfate / isoferulic acid sulfate	$[M-H]^-$	273.0073	0.97	0.97	-0.59	0.04	2
1385	$C_9H_{12}ClN_2O_4P$	--	$[M-H]^-$	277.0150	6.66	-0.17	-0.23	0.03	4
1438	$C_{11}H_{12}O_7S$	dihydroxy-phenyl-gamma-valerolactone-sulfate	$[M-H]^-$	287.0231	1.03	-0.04	-0.6	0.02	3
1439	$C_{13}H_{12}N_4S_2$	--	$[M-H]^-$	287.0438	6.67	--	-0.2	0.04	4
1464	$C_7H_4Br_2O_3$	dibromo-hy-droxybenzoic acid	$[M-H]^-$	292.8455	1.19	0.28	-0.77	0.03	2
1475	$C_7H_6Br_2OS$	dibromo-me-thylthio-phenyl-etha-none	$[M-H]^-$	294.8433	1.19	0.34	-0.78	0.03	2
1483	$C_{17}H_{28}O_4$	Terpenoid	$[M-H]^-$	295.1913	1.26	-0.61	-0.57	0.03	2
1499	$C_{16}H_{28}O_5$	FA(16:2; O3)	$[M-H]^-$	299.1862	1.26	-0.53	-0.41	0.03	2
1502	$C_8H_5N_3O_6P_2$	--	$[M-H]^-$	299.9577	5.17	-1.20	-0.30	0.04	4
1507	$C_{16}H_{30}O_5$	FA(16:1; O3)	$[M-H]^-$	301.2018	1.26	-0.68	-0.68	0.03	2
1641	$C_{15}H_{10}O_7S$	daidzein-sul-fate	$[M-H]^-$	333.0071	1.29	-1.02	-0.59	0.03	2
**1742**	$\mathbf{C_{24}H_{38}O_2}$	**Bile acids and derivatives**	$\mathbf{[M-H]^-}$	**357.2796**	**1.17**	**-0.69**	**0.54**	**0.03**	**2**
**1766**	$\mathbf{C_{20}H_{30}O_6}$	**hydroxy-12-oxo-eicosa-trienedioic acid or trihydroxy-12-keto-eico-satetraenoic acid**	$\mathbf{[M-H]^-}$	**365.1966**	**1.19**	**-0.94**	**-0.44**	**0.02**	**2**
1774	$C_{23}H_{46}O_3$	FA(23:0; O)	$[M-H]^-$	369.3371	1.19	-0.88	-0.47	0.04	2
**1780**	$\mathbf{C_{24}H_{38}O_3}$	**Bile acids and derivatives**	$\mathbf{[M-H]^-}$	**373.2744**	**1.20**	**-1.19**	**0.49**	**0.05**	**2**
1788	$C_{18}H_{32}O_6S$	--	$[M-H]^-$	375.1843	1.34	--	-0.79	0.03	
**1807**	$\mathbf{C_{20}H_{30}O_7}$	**tetrahydroxy-12-oxo-tet-raenoic acid**	$\mathbf{[M-H]^-}$	**381.1917**	**1.10**	**-0.51**	**-0.18**	**0.04**	**2**
1811	$C_{24}H_{24}N_4O$	--	$[M-H]^-$	383.1891	1.16	3.82	-0.18	0.04	4
1858	$C_{21}H_{22}O_9$	flavonoid o-glycoside	$[M-H]^-$	417.1186	4.27	-1.06	-0.55	0.03	2
1872	$C_{19}H_{36}N_{10}S$	--	$[M-H]^-$	435.2775	1.17	--	-0.69	0.02	4
1878	$C_{21}H_{43}N_3OP_2S$	--	$[M-H]^-$	446.2534	1.19	1.09	-0.36	0.04	4
1879	$C_{27}H_{36}N_4O_2$	--	$[M-H]^-$	447.2770	1.19	1.11	-0.62	0.04	4
1953	$C_{32}H_{58}N_6O_2$	--	$[M-H]^-$	557.4540	1.24	-1.47	-0.49	0.04	4
1961	$C_{26}H_{50}NO_7P$	LPC(18:2)	$[M+H_2CO_2-H]^-$	610.3352	4.96	-1.75	-0.24	0.05	2

Table S8. Pathway analysis results obtained from Lipid Pathway Enrichment Analysis (LIPEA) of RP data.

Pathway name	Pathway lipids	Converted lipids (number)	Converted lipids (%)	Converted lipids (list)
Steroid biosynthesis	41	1	4.1	C02530
Sphingolipid metabolism	21	3	12.5	C00195, C12126, C00550
Glycosylphosphatidylinositol (GPI)-anchor biosynthesis	3	2	8.3	C01194, C00350
Glycerophospholipid metabolism	26	8	33.3	C02737, C04438, C05973, C04230, C00157, C00350, C04233, C01194
alpha-Linolenic acid metabolism	23	1	4.1	C00157
Inositol phosphate metabolism	9	1	4.1	C01194
Sphingolipid signaling pathway	9	3	12.5	C00195, C12126, C00550
Ether lipid metabolism	16	2	8.3	C04475, C05212
Glycine, serine and threonine metabolism	3	1	4.1	C02737
Arachidonic acid metabolism	75	1	4.1	C00157
Autophagy - other	3	2	8.3	C01194, C00350
Linoleic acid metabolism	25	1	4.1	C00157
Autophagy - animal	4	2	8.3	C01194, C00350
Necroptosis	4	2	8.3	C00195, C00550
Neurotrophin signaling pathway	3	1	4.1	C00195
Phosphatidylinositol signaling system	11	1	4.1	C01194
Adipocytokine signaling pathway	3	1	4.1	C00195
Ferroptosis	11	3	12.5	C21480, C21481, C21484
Retrograde endocannabinoid signaling	8	2	8.3	C00157, C00350
Ovarian steroidogenesis	18	1	4.1	C02530
Fat digestion and absorption	8	1	4.1	C02530
AGE-RAGE signaling pathway in diabetic complications	2	1	4.1	C00195
Insulin resistance	4	1	4.1	C00195
Leishmaniasis	4	2	8.3	C00195, C02737
Tuberculosis	5	1	4.1	C01194
Vitamin digestion and absorption	15	1	4.1	C02530
Cholesterol metabolism	8	1	4.1	C02530
Amoebiasis	4	1	4.1	C02737
Kaposi's sarcoma-associated herpesvirus infection	3	1	4.1	C00350
Systemic lupus erythematosus	1	1	4.1	C02737
Choline metabolism in cancer	5	2	8.3	C04230, C00157
Bile secretion	25	1	4.1	C02530

1. Horai, H.; Arita, M.; Kanaya, S.; Nihei, Y.; Ikeda, T.; Suwa, K.; Ojima, Y.; Tanaka, K.; Tanaka, S.; Aoshima, K.; Oda, Y.; Kakazu, Y.; Kusano, M.; Tohge, T.; Matsuda, F.; Sawada, Y.; Hirai, M. Y.; Nakanishi, H.; Ikeda, K.; Akimoto, N.; Maoka, T.; Takahashi, H.; Ara, T.; Sakurai, N.; Suzuki, H.; Shibata, D.; Neumann, S.; Iida, T.; Tanaka, K.; Funatsu, K.; Matsuura, F.; Soga, T.; Taguchi, R.; Saito, K.; Nishioka, T., MassBank: a public repository for sharing mass spectral data for life sciences. *J Mass Spectrom* **2010,** *45* (7), 703-14.
